# Thyroid Activating Enzyme, Deiodinase II Is Required for Photoreceptor Function in the Mouse Model of Retinopathy of Prematurity

**DOI:** 10.1167/iovs.61.13.36

**Published:** 2020-11-25

**Authors:** Onkar B. Sawant, Vijay K. Jidigam, Kenya Wilcots, Rebecca D. Fuller, Ivy Samuels, Sujata Rao

**Affiliations:** 1 Department of Ophthalmic Research, Cole Eye Institute, Cleveland Clinic, Cleveland, Ohio, United States; 2 Research Service, Louis Stokes Cleveland Veterans Affairs Medical Center, Cleveland, Ohio, United States; 3 Department of Ophthalmology, Cleveland Clinic Lerner College of Medicine of Case Western Reserve University, Cleveland, Ohio, United States; 4 Department of Chemistry, Cleveland State University, Cleveland, Ohio, United States; 5 Eversight, Cleveland, Ohio, United States

**Keywords:** ROP, OIR, adult cone photoreceptor, Dio2, thyroid hormone

## Abstract

**Purpose:**

Retinopathy of prematurity (ROP) is a severe complication of premature infants, leading to vision loss when untreated. Presently, the molecular mechanisms underlying ROP are still far from being clearly understood. This study sought to investigate whether thyroid hormone (TH) signaling contributes to the neuropathology of ROP using the mouse model of ROP to evaluate longitudinal photoreceptor function.

**Methods:**

Animals were exposed to hyperoxia from P7 to P12 to induce retinopathy, thereafter the animals were returned to room air (normoxia). The thyroid-activating enzyme type 2 deiodinases (Dio2) knockout (KO) mice and the littermate controls that were exposed to hyperoxia or maintained in room air and were then analyzed. The retinal function was evaluated using electroretinograms (ERGs) at three and seven weeks followed by histologic assessments with neuronal markers to detect cellular changes in the retina. Rhodopsin protein levels were measured to validate the results obtained from the immunofluorescence analyses.

**Results:**

In the ROP group, the photoreceptor ERG responses are considerably lower both in the control and the *Dio2 KO* animals at P23 compared to the non-ROP group. In agreement with the ERG responses, loss of Dio2 results in mislocalized cone nuclei, and abnormal rod bipolar cell dendrites extending into the outer nuclear layer. The retinal function is compromised in the adult *Dio2 KO* animals, although the cellular changes are less severe. Despite the reduction in scotopic a-wave amplitudes, rhodopsin levels are similar in the adult mice, across all genotypes irrespective of exposure to hyperoxia.

**Conclusions:**

Using the mouse model of ROP, we show that loss of Dio2 exacerbates the effects of hyperoxia-induced retinal deficits that persist in the adults. Our data suggest that aberrant Dio2/TH signaling is an important factor in the pathophysiology of the visual dysfunction observed in the oxygen-induced retinopathy model of ROP.

Thyroid hormones are essential for gestational and postnatal neurodevelopment, playing an important role in neurogenesis, proliferation and migration, dendritic formation, synaptic connectivity, and myelination.^1^^−^^5^ Thyroid hormone (TH) is predominantly secreted into circulation as an inactive prohormone, thyroxine (T4). T4 enters the cell via membrane transporters such as MCT8, MCT10, OATP1C1, and L-type amino acid transporters.^6^^−^^10^ The regulation of T4 is controlled by the activity of two enzymes; type 2 iodothyronine deiodinase (Dio2) and type 3 iodothyronine deiodinase (Dio3). In the target tissue, the majority of cytoplasmic T4 is converted enzymatically into the active tri-iodothyronine (T3) by Dio2. Dio3 modulates T4 and T3 by converting them into reverse T3 and T2, respectively.^10^ T3 generated by Dio2 contributes to the nuclear pool of T3 and in combination with the TH receptors mediates transcriptional regulation of downstream targets.^11^

Thyroid dysfunction during pregnancy and the neonatal period is associated with fetal neuronal retardation and overall fetal growth restriction. During gestation, the fetus is largely dependent on the maternal TH supply until the fetal hypothyroid-pituitary-thyroid (HPT) axis starts developing in the third trimester.^12^ Thus hypothyroidism is a common occurrence in preterm infants, and more than 50% of the low–birth weight preterm infants have some type of thyroid dysfunction.^13^ Many preterm infants can also develop retinopathy of prematurity (ROP). ROP is a vasoproliferative disease of the developing retina and is a major cause of blindness in premature babies. Although the incidence of ROP is approximately 60%, the rates can vary depending on the birth and survival rates of premature infants in different countries, their birth weight, and gestational age.^14^^,^^15^

The age of ROP onset coincides with the rapid developmental increase in photoreceptor outer segment length, opsin content, circulating TH levels, retinal expression of TH transporter Mct8, and TH-activating enzyme Dio2.

We and others have shown that the levels of TH and intracellular components of TH signaling play a crucial role in the genesis, development, maintenance, and function of the photoreceptors.^16^^−^^18^ Although clinical studies have repeatedly shown that photoreceptor function is affected in the ROP patients,^19^^−^^23^ minimal efforts have been made to understand the role of TH and TH signaling components in ROP pathology.^24^ The goal of this study is to investigate whether the thyroid-activating enzyme, Dio2 contributes to the pathophysiology of ROP using the oxygen-induced retinopathy (OIR) model. The mouse model of OIR is widely used to study the signaling pathways that are important for the development and progression of ROP. Using this model, we show that the Dio2 signaling pathway contributes to ROP pathology. Accordingly, in the *Dio2 KO* animals exposed to hyperoxia, there is a significant reduction in retinal response to light flashes under both scotopic and photopic conditions. Moreover, a longitudinal assessment of retinal morphology and function suggest that retinal function is not fully restored in the loss of *Dio2 KO* adult animals. Our data demonstrates that thyroid hormone/Dio2 signaling contributes to ROP pathology.

## Methods

### Animals and OIR

All animal studies were approved by the Institutional Animal Care and Use Committee of the Cleveland Clinic and conformed to current National Institutes of Health guidelines (Guide for the Care and Use of Laboratory Animals in Research, 8th ed., 2011). The animals were cared for in accordance with the ARVO Statement for the Use of Animals in Ophthalmic and Vision Research. Transgenic mice with the Dio2 gene deletion (Stock No: 018985) were purchased from the Jackson Laboratory (Bar Harbor, ME, USA). The ROP model used in this study is based on the protocol established by Smith et al.^25^ In brief, heterozygous nursing mothers (that were mated to males either heterozygote or homozygous for the *Dio2* allele) and their pups were placed in 75% oxygen (hyperoxia) for five days (P7 through P12, date of birth is P0). A plexiglass incubator containing an oxygen sensor and feedback system (ProOx; Biospherix, Ltd., Lacona, NY, USA) was used to ensure continuous hyperoxic conditions. The *Dio2* heterozygotes are indistinguishable from the *Dio2* wild type animals in their ERG responses, as well as for immunohistochemistry (Supplementary Figs. S1 and S2). However, the numbers of wild-type (WT) litter mate controls were very low; hence, Dio2^+/−^ are used as controls for the analysis. Animals were returned to the normal oxygen conditions (normoxia) at P12. Mice were euthanized using isoflurane (Piramal Critical Care, Inc., Bethlehem, PA, USA) in an appropriate inhalation chamber.

### IR Western Blot

Eyes were dissected in cold PBS and retinas were lysed in lysis buffer (100 µL per retina) composed of 20 mM Tris-HCl, pH 8, 150 mM NaCl, 2.5 mM EDTA, 10% glycerol, 0.5% Triton X-100, 0.01% Nonidet P-40 substitute, protease inhibitor and phosphatase inhibitor tablets (Roche Diagnostics, Indianapolis, IN, USA). Blots were probed with antibodies against rhodopsin (ab98887; Abcam, Cambridge, MA, USA) and β-actin (4970L; Cell Signaling Technology, Danvers, MA, USA). Immunoblots were visualized using IRDye 800CW donkey anti-rabbit secondary antibody (925-32213; Li-Cor Biosciences, Lincoln, NE, USA) and IRDye 680CW donkey anti-mouse secondary antibody. Membranes were scanned with an Odyssey infrared scanner (Li-Cor Biosciences). Densitometric analysis was performed using image studio version 5.2 (Li-Cor Biosciences). For the quantitation, 4 independent proteins blots were used and each lane represents retinal lysates from different animals. Six to eight animals were used per genotype for the normoxia and hyperoxia conditions.

### Immunohistochemistry

For cryosections, enucleated eyes were fixed in 4% PFA for 90 minutes at room temperature (RT), washed in PBS, cryoprotected using sucrose gradient (15%, 30%), and kept overnight in 30% sucrose at 4°C. Eyes were mounted in the mounting media via dorsoventral orientation as described by Wagner et al.^26^ Sections were washed in PBS, permeabilized for five minutes using 1% Triton X-100 and blocked for one hour at RT with PBS containing 3% BSA and 0.03% Triton X-100. Cryosections were incubated with the following primary antibodies: S-opsin (1:500) (AB5405; Millipore, Billerica, MA, USA), Caspase-3 (1:200) (ab559565; BD Pharmingen, San Jose, CA, USA), Protein Kinase C alpha (PKCα) (1:1000) (sc208; Santa Cruz Biotechnology, Dallas, TX, USA), Recoverin (1:500) (AB5585; Millipore), Cone Arrestin (1:200) (Millipore) and Rhodopsin (1:5000) (ab98887; Abcam, Cambridge, MA, USA) overnight at 4°C. Appropriate Alexa Fluor conjugated secondary antibodies were used for labeling. (Life Technologies, Carlsbad, CA, USA). Images were acquired using a Leica laser scanning confocal microscope (TCSSP2; Leica, Exton, PA, USA). For cone nuclear counts, multiple images were acquired for each section from the middorsal and midventral regions. Images were divided equally divided into three zones, and total number of nuclei per zone were counted. All nuclear counts were normalized for 200 µm/section. For each genotype and condition, multiple sections through the optic nerve were labeled and were averaged for n = 1.

### Electroretinography

In brief, at either P23 or seven weeks of age, mice were dark-adapted overnight and then prepared for electroretinography in red light. Mice were anesthetized using ketamine (40 mg/kg) and xylazine (8 mg/kg), pupils were dilated using 1% tropicamide, 2.5% phenylephrine hydrochloride, and 1% cyclopentolate (Akorn Inc., Lake Forest, IL, USA). Eyes were anesthetized with 0.5% proparacaine hydrochloride (Akorn, Inc.). ERGs were recorded using an Espion E3 ColorDome Full field Ganzfeld (Diagnosys, LLC, Lowell, MA, USA). Mice were placed on a temperature-regulated heating pad and scotopic responses were recorded in the dark in response to a green light-emitting diode (LED) stimulus at 520 nm, half-bandwidth of 35 nm at increasing intensities of −3.6, −3, −2.4, −1.8, −1.2, −0.6, 0, 0.6, 1.4, and 1.9 log cd ∙ s/m^2^. Sampling frequency was 1202 Hz. To measure cone responses, rods were first saturated with a steady-state green + blue stimulus (10 cd/m^2^) for seven minutes and a green LED (peak at 520 nm, half-bandwidth of 35 nm) was superimposed on the steady-state background at increasing intensities of −0.6, 0, 0.6, 1.4, and 1.9 log (P) cd/m^2^. Sampling frequency was 100 Hz with 20 responses averaged per recorded trace. After the conclusion of the recording session, the eyes were coated with Polycin ophthalmic ointment (Perrigo, Minneapolis, MN, USA), and the mice were returned to clean cages on a heating pad until awake. Amplitude of the scotopic a-wave was measured at 8 ms. Amplitude of the scotopic b-wave was measured by summing the maximum amplitude of the waveform following the oscillatory potentials (between 40 and 150 ms) with the calculated amplitude at each intensity. Amplitude of the photopic a-wave and b-wave was determined in the same manner as the scotopic response. Oscillatory potentials (OPs) were filtered from the strobe-flash response, and amplitudes were measured from the trough to the peak of each potential from the filtered waveform.

### Statistical Analysis

Statistical significance was determined using GraphPad Prism 8. The ERG and cone density data were analyzed using two-way ANOVA followed by pairwise multiple comparison using Tukey test and Bonferroni post-test. Significance is denoted as *P < 0.05, **P < 0.01, ***P < 0.001, ****P < 0.0001.

## Results

### *Dio2 KO* Mice Exposed to Hyperoxia Display Severe ERG Defects at P23

**Figure 1.**
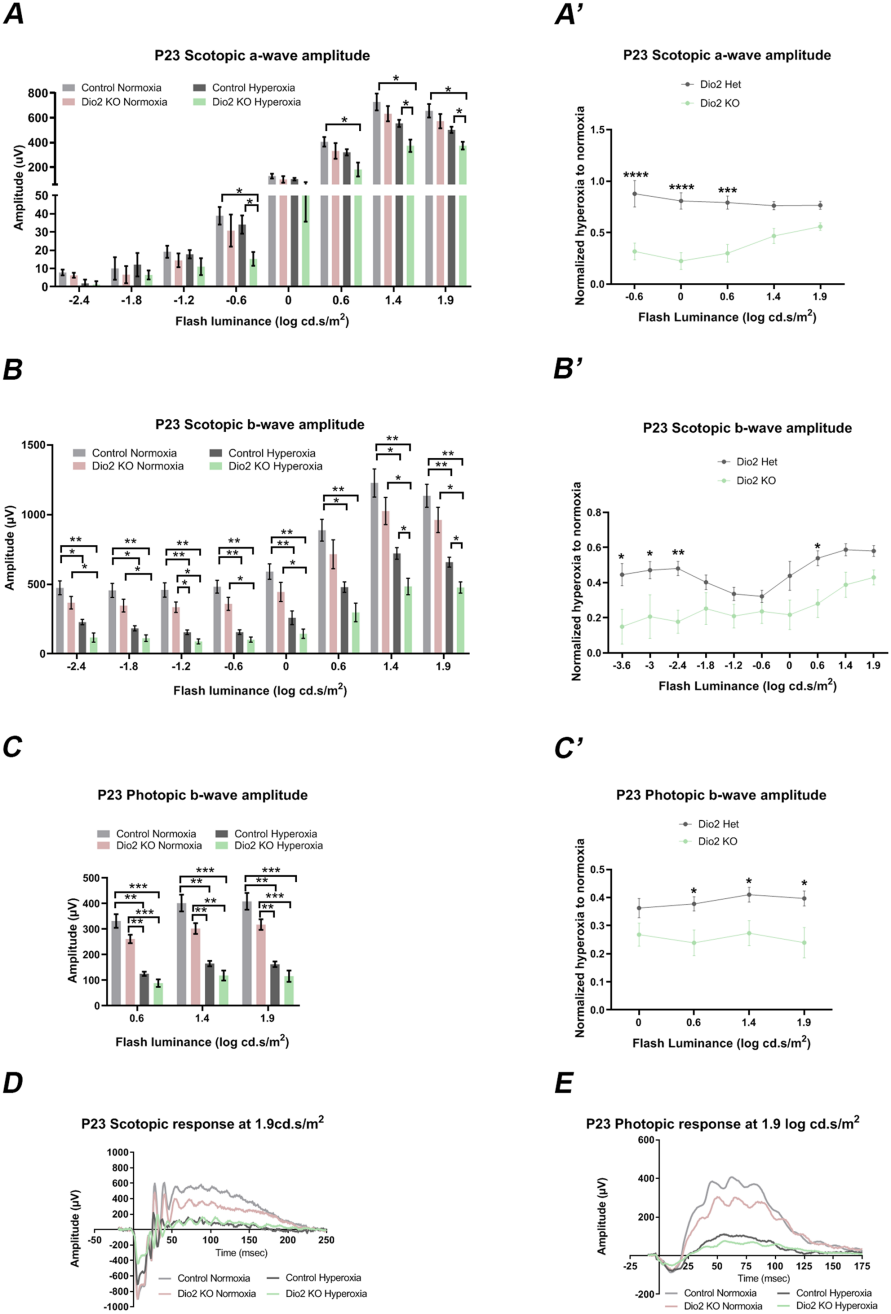
Loss of *Dio2* exacerbates the visual dysfunction induced by hyperoxia. (**A, B**) Quantitation of ERG a- and b-wave amplitudes at multiple light intensities in control (*Dio2^+/^*^−^) and *Dio2 KO* (*Dio2^-/-^*) mice under normoxia and hyperoxia conditions. A-wave amplitudes are not significantly different between control and *Dio2 KO* animals at low flash intensities; however, at higher intensities (≥ −0.6 log cd ∙ s/m^2^) a-wave amplitudes are significantly lower in *Dio2 KO* animals exposed to hyperoxia at flash intensities except at 0 log cd ∙ s/m^2^. Scotopic b-wave amplitudes in the *Dio2 KO* animals were significantly lower than the control animals under both normoxia and hyperoxia conditions. (**A’, B’**) Amplitudes of scotopic a- and b-wave under hyperoxic conditions normalized to normoxia for the control and *Dio2 KO* to demonstrate the overall effect of hyperoxia on each genotype. (**C**) Amplitudes of the photopic ERG b-wave recorded from *Dio2 KO* and controls compared to their normoxia cohorts, at different flash intensities indicating the significant effect of hyperoxia on cone responses (**C’**) amplitudes were normalized to its respective normoxic genotype to further illustrate that hyperoxia significantly affects cone responses in the *Dio2 KO* compared to the controls. (**D**) Representative scotopic ERG responses recorded in response to 1.9 log cd ∙ s/m^2^ white flash stimulus from the controls and *Dio2 KO* animals under normoxic and hyperoxic conditions. (**E**) Representative photopic ERG responses recorded in control and *Dio2 KO* animals under normoxia and hyperoxia conditions (n = 4–10). *P < 0.05, **P < 0.01, ***P < 0.001, ****P < 0.0001. Data were analyzed using two-way ANOVA and pairwise post-hoc comparisons were performed using Tukey's method and Bonferroni post-test. *Error bars:* ±SEM.

We previously reported that Dio2 is required for cone photoreceptor function, but does not affect rod responses.^18^ To determine whether Dio2 contributes to the pathology of ROP, we performed ERG analysis on *Dio2 KO* and litter mate control animals maintained in normoxia or exposed to hyperoxia for five days. To evaluate photoreceptor function, we measured dark-adapted (scotopic) a-wave amplitude at P23 (Figs. 1A, 1D). Under normoxic conditions, the scotopic a-wave responses between the control (*Dio2^+/^*^−^) and *Dio2 KO* were similar. However, in animals that were exposed to hyperoxia, photoreceptor-derived responses were significantly lower (*P* < 0.05) in *Dio2 KO* animals compared to the normoxia and hyperoxia controls at flash intensities −0.6, 1.4 and 1.9 log cd ∙ s/m^2^ (Fig. 1A).

Next we investigated if scotopic b-wave amplitudes are altered by hyperoxia. Overall, exposure to hyperoxia results in a significant reduction in the scotopic b-wave amplitudes independent of the genotypes of the animals (Fig. 1B). However, consistently the b-wave amplitudes in the *Dio2 KO* animals from hyperoxia group were significantly lower when compared to the *Dio2 KO* animals under normoxic conditions. Interestingly, at high flash intensities (1.4 and 1.9 log cd ∙ s/m^2^) there is a further significant reduction (*P* < 0.05) in the b-wave amplitudes recorded from the *Dio2 KO* hyperoxia animals compared to the hyperoxia controls. Under dark-adapted conditions, the b-wave response is largely indicative of rod bipolar cell response.^27^ This would suggest that hyperoxia is severely affecting the bipolar cells. We re-plotted the data differently to determine whether hyperoxia resulted in a more severe phenotype in the *Dio2 KO* animals compared to the controls (Figs. 1A’, 1B’, 1C’). For the scotopic a wave responses, at flash luminance of −0.6, 0 and 0.6 log cd ∙ s/m^2^, loss of Dio2 exacerbates the effects of hyperoxia significantly (*P* < 0.0006, Supplementary Table S1). Similarly, for scotopic b-wave responses, there is a significant effect of hyperoxia on *Dio2 KO* compared to the controls at lower flash luminance (*P* < 0.05 at −3.6, −3 log cd ∙ s/m^2^, *P* < 0.01 at −2.4 log cd ∙ s/m^2^) and at 0.6 log cd ∙ s/m^2^ (*P* < 0.05). Thus hyperoxia drastically alters the scotopic visual responses in the *Dio2 KO* animals and appears to compromise the bipolar cell responses far more severely. Furthermore, the light-adapted (photopic) responses are also affected by hyperoxia, as the photopic b-wave amplitudes are reduced in both control animals (normoxia vs. hyperoxia) and *Dio2 KO* (normoxia vs. hyperoxia) (Figs. 1C, 1C’, 1E). It is important to note that the effect of hyperoxia in the *Dio2 KO* animals is more severe compared to the control at higher flash luminance (*P* < 0.05 at 0.6 and 1.4, 1.9 log cd ∙ s/m^2^, Fig. 1C’). Taken together, these results indicate that at P23, retinal function is severely compromised by hyperoxia in the *Dio2 KO* animals.

### Loss of *Dio2* Exacerbates Hyperoxia-Induced Cellular Changes in the Retina

**Figure 2.**
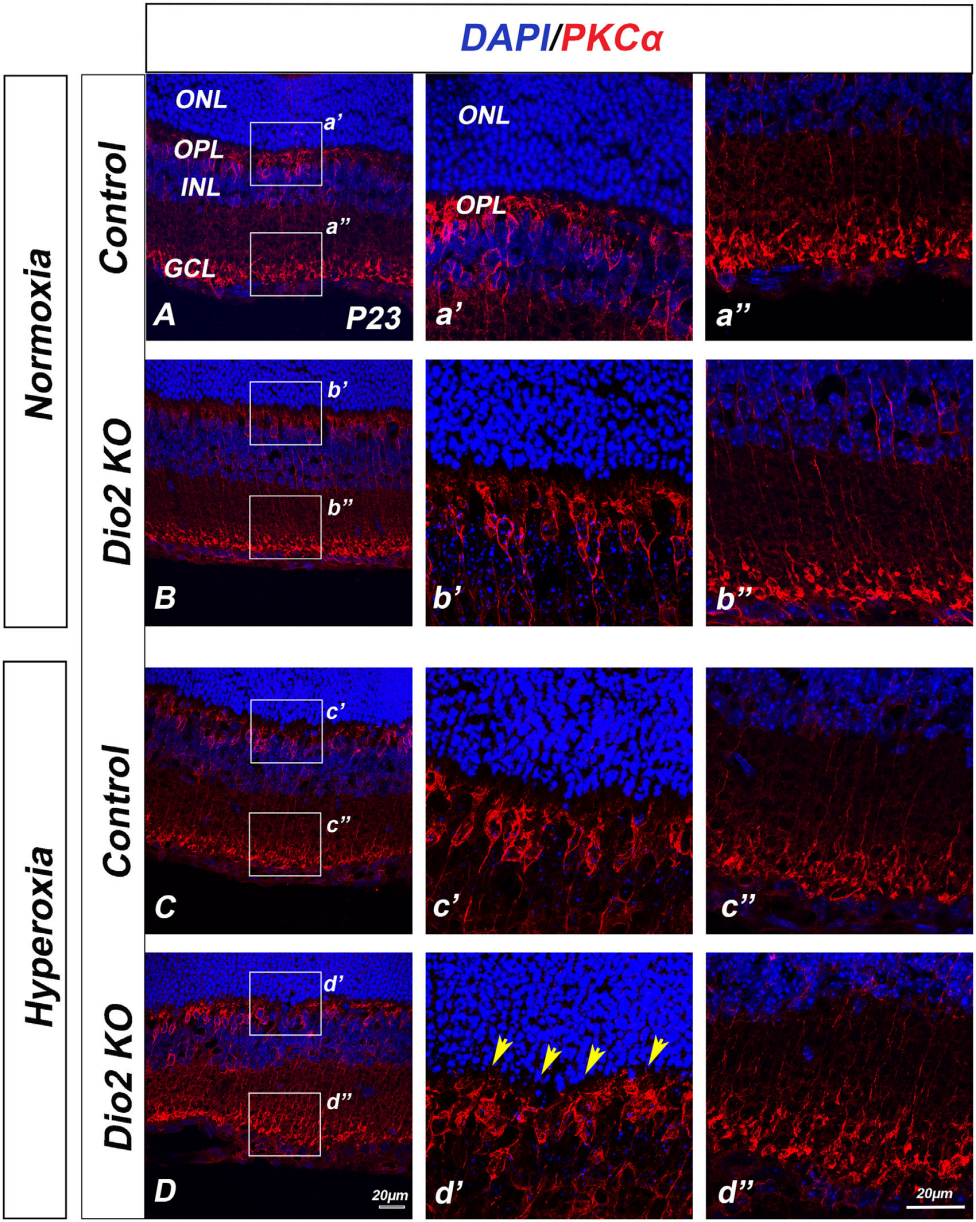
Morphological defects in Rod bipolar dendrites in the *Dio2 KO* hyperoxia. Immunostaining of rod bipolar cells (RBC) in P23 mouse retinal sections. (**A–D**) RBCs are stained with anti- PKCα in *red* and DAPI in *blue*. Magnified images of the indicated regions (*white square*) from controls (**a’ & a”, c’ & c”**) and *Dio2 KO* (**b’ & b”, d’ & d”**) under normoxia (**A–b”**) and hyperoxia (**C–d”**) conditions. (**a’, b’, c’, d’**) Represent the rod bipolar dendritic processes that extend into the OPL whereas (**a”, b”, c”, d”**) indicate the PKCα staining of RBC toward GCL layer. Controls are *Dio2^+/^*^−^. *Yellow arrow heads* indicating the dendrites crossing the OPL and reaching ONL (n = 3). GCL, ganglion cell layer; OPL, outer plexiform layer.

**Figure 3.**
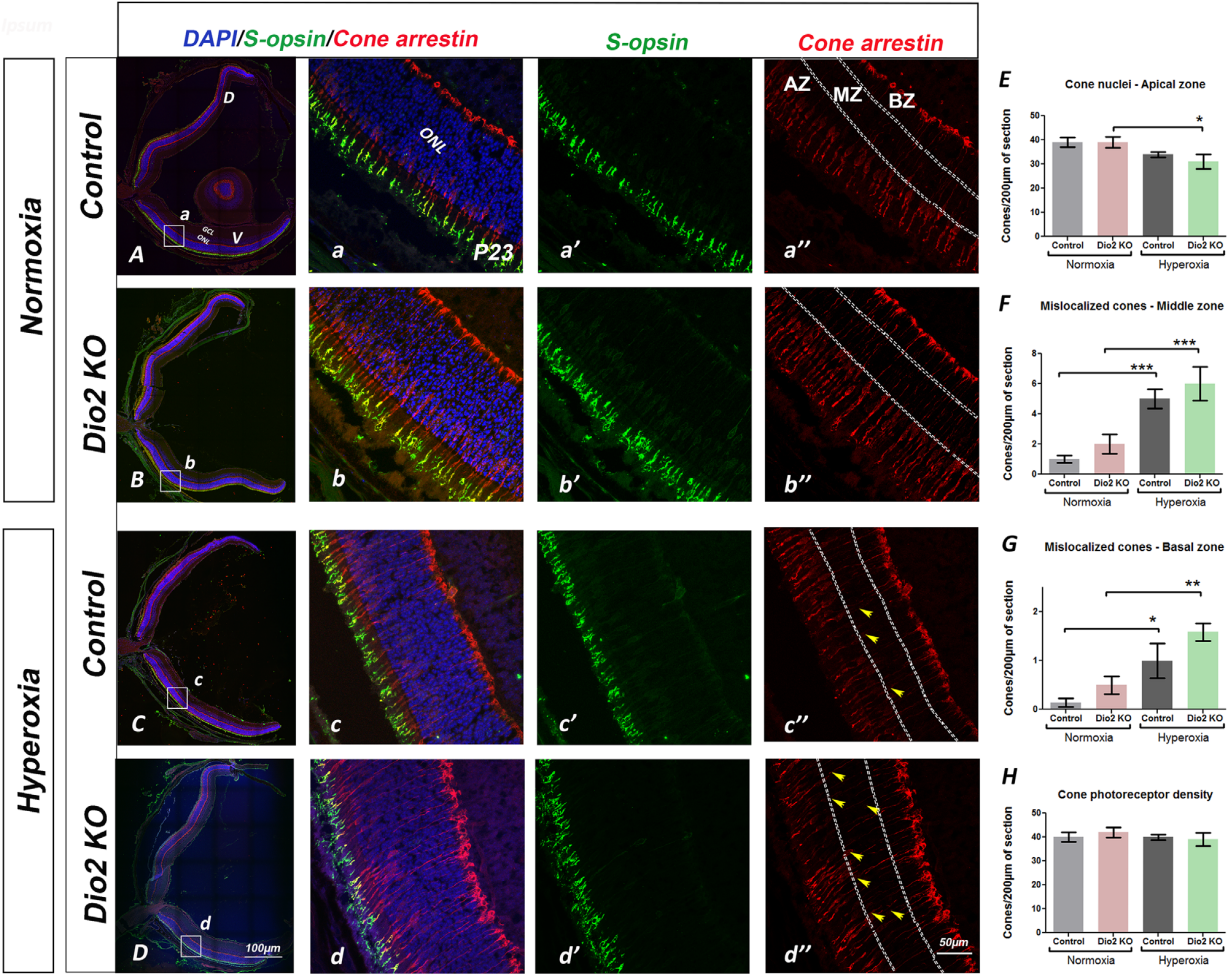
Mislocalized cone nuclei in *Dio2 KO* animals. (**A–D”**) Cryosections from P23 animals labeled with S-opsin (*green*) and Cone Arrestin (red) in *Dio2^+/^*^−^ (**A–A”, C–C”**) and *Dio2 KO* (**B–B”, D–D”**) under normoxia (**A–B”**) and hyperoxia (**C–D”**). (**E–G**) quantitation of the mislocalized cone nuclei in the apical (**E**), middle (**F**), and basal zones (**G**). (**H**) Graph indicates the total cone density between different conditions and genotypes. Note that the cone photoreceptor density is not significantly different between the groups. *Yellow arrowheads* indicate mislocalized cones (n = 3–7). *P < 0.05, **P < 0.01, ***P < 0.001. Data were analyzed using two-way ANOVA, and pairwise post-hoc comparisons were performed using Fisher LSD method. *Error bars:* ±SEM.

It has been previously shown that hyperoxia does not affect the outer nuclear layer (ONL) thickness, suggesting that hyperoxia does not result in cellular loss.^28^ However, the deficits in the visual responses could be a result of cellular changes that may not alter the overall numbers of the photoreceptors but could still affect photo transduction. To determine if hyperoxia causes increased cell death in the *Dio2 KO* animals, retinal sections of each genotype were labeled with active Caspase-3. At P23, very few Caspase-3 positive nuclei can be detected either in the control or in the *Dio2 KO* retina (Supplementary Fig. S3), thus excluding cell loss as the underlying cause of the observed ERG phenotypes. To investigate whether underlying changes in cellular morphology of the outer retinal cells are the cause of impaired retinal function, we labeled the retina with markers for photoreceptors and bipolar cells. At P23, rhodopsin immunoreactivity between the genotypes and the various conditions appears to be similar (Supplementary Fig. S4). This is surprising given that we see a difference in scotopic a-wave responses under hyperoxia conditions. However, the immunofluorescence staining may not be sensitive enough to detect subtle changes in rhodopsin levels, so we cannot rule out that possibility. Alternatively, there could be changes in other rod photoreceptor proteins which can result in compromised rod function. To assess whether rod bipolar cell morphology was altered, retinal sections were labeled with PKC-alpha. (Fig. 2). Within the normoxia group, the boundary between the ONL and the rod bipolar cells (RBC) dendrites is maintained with most of the dendrites within the outer plexiform layer (Figs. 2A, 2B, 2C). However, in the hyperoxia group the RBCs extend several of the dendrites within the ONL and RBCs are more severely affected by the loss of Dio2 (Figs. 2D). Unlike the RBCs, the cone bipolar cells labeled with recoverin did not show any change in number or location (Supplementary Fig. S5). Though the cone bipolar cells were not affected, the cone photoreceptor nuclei appear to be mislocalized in the *Dio2 KO* retina (Figs. 3A–3D). To quantitatively analyze this phenotype, we arbitrarily divided the ONL into three different zones (apical, middle and basal) and counted the number of cone arrestin–positive nuclei within each zone (details provided in the Methods section). The cones were also co-labeled with S-opsin, to assess if the cone opsins are also mislocalized. There wasn't any detectable difference in the cone opsin distribution except that in the *Dio2 KO* retina, as previously reported, the S cones are present in the dorsal region.^18^ The spatial distribution was not affected by hyperoxia. Generally, in the normoxic control retinas, almost all the cone nuclei are located in the apical zone of the ONL (Figs. 3E, 3F, 3G). In the animals that were exposed to hyperoxia, we observed a significant increase in the numbers of cone nuclei in the middle (Fig. 3F) and basal (Fig. 3G) zones and a concomitant decrease in the apical zone (Fig. 3E). We also quantitated the cone density by counting the total number of cones /200micron of the section (Fig. 3H). Cone density among the different groups remained the same suggesting that the cones are not dying but their nuclear positioning is affected by the loss of Dio2. Overall, the cellular analysis data further demonstrates that under hyperoxic conditions, Dio2 signaling plays an important role for the correct development of the rod bipolar cells and the cone photoreceptors but not cone bipolar cells.

### *Dio2* is Required for Long-Term Recovery from the Hyperoxia-Induced Damage

**Figure 4.**
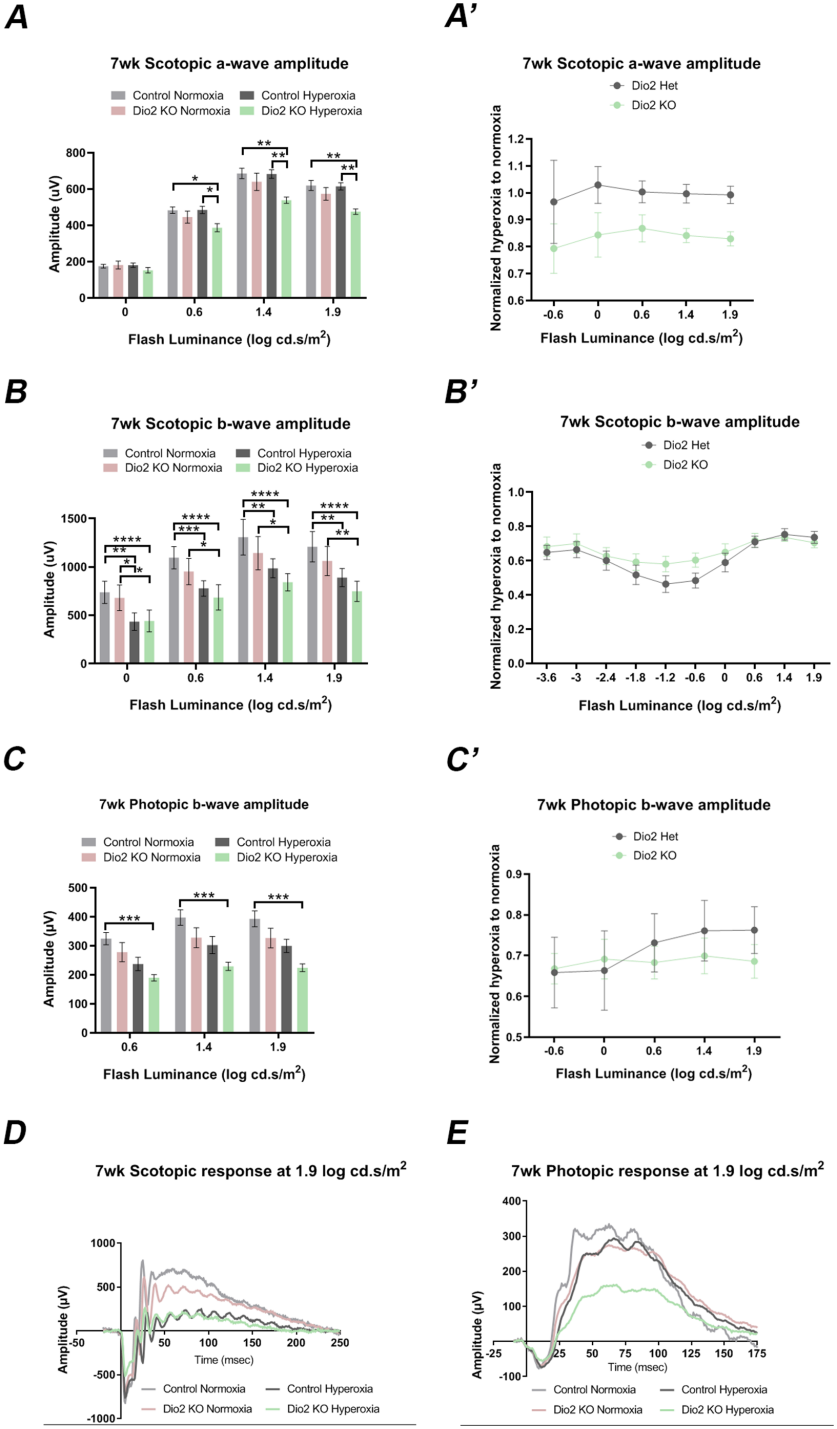
ERG responses recorded from adult animals demonstrating the long-term effects of hyperoxia on retinal function. (**A, B**) Amplitudes of the recorded scotopic a-wave (**A**) and b-wave responses (**B**) from control (*Dio2^+/^*^−^) and *Dio2 KO* animals under different conditions. Scotopic a-wave amplitudes were significantly reduced in *Dio2 KO* animals from the hyperoxia group at the flash intensities > 0.6 log cd ∙ s/m^2^ when compared to other groups. Scotopic b-wave amplitudes were significantly reduced in the hyperoxia independent of the genotype. (**A’, B’**) Normalized amplitudes of the scotopic a and b-wave to indicate the overall effect of hyperoxia on genotypes. In the adults, there is a long-lasting effect of hyperoxia on rod a-wave amplitude in the *Dio2 KO* animals (**A’**), but the b-wave wave responses appear to recover in the *Dio2 KO* animals compared to the controls (**B’**). (**C, C’**) Photopic b-wave amplitudes measured from the control and *Dio2 KO* animals under normoxia and hyperoxia. The cone photoreceptor function is not completely restored in either control or *Dio2 KO* animals that were exposed to hyperoxia though at 1.9 log cd ∙ s/m^2^, the Dio2 scotopic b-wave amplitudes are significantly lower compared to all the groups (**C**). (**C’**) Normalized photopic amplitudes to illustrate the differential effects of hyperoxia on the genotypes. Note that although overall hyperoxia results in reduction in cone b-wave amplitudes, the effect is not significant. (**D**) Representative scotopic ERG responses recorded from the control and *Dio2 KO* animals in normoxia and hyperoxia conditions. (**E**) Representative photopic ERG responses recorded at 1.9 log cd ∙ s/m^2^ flash intensity between the control and *Dio2 KO* animals in normoxia and hyperoxia conditions (n = 5–11). *P < 0.05, **P < 0.01, ***P < 0.001, ****P < 0.0001. Data were analyzed using two-way ANOVA, and pairwise post-hoc comparisons were performed using Tukey's method and Bonferroni post-test. *Error bars:* ±SEM.

It has been reported that hyperoxia does not result in long-term changes in photoreceptor function^28^ despite the severe cellular changes that are detected in the animals at P23. To determine whether *Dio2 KO* animals recover from the effects of hyperoxia, animals were maintained in room air for 5 weeks after exposure to hyperoxia and ERGs were recorded. Scotopic a-wave amplitudes in the *Dio2 KO* animals from the hyperoxia group were significantly reduced (Supplementary Table S2) compared to hyperoxia controls (*Dio2^+/^*^−^) at flash luminance 0.6 (*P* < 0.05), 1.4 and 1.9 log cd ∙ s/m^2^ (*P* < 0.005) (Fig. 4A). No significant differences were observed between groups for scotopic a-wave amplitude for flash intensities ≤ 0.0 log cd ∙ s/m^2^ (Fig. 4A). Moreover, at flash intensity 0.6, 1.4 and 1.9 log cd ∙ s/m^2^ (*P* < 0.05) there is a significant reduction in a-wave amplitude measured from the hyperoxia *Dio2 KO* compared to the normoxia animals. In general, the scotopic ERG responses recorded from the *Dio2 KO* group are lower compared to the controls at higher intensities. The origin of such a mixed response is difficult to explain, but it is possible that the rod photoreceptors do not recover completely after exposure to hyperoxia or that the cone response elicited by the high intensity flashes reveals a role for the cones. Unlike the a-wave response, the scotopic b-wave responses were significantly reduced at all intensities tested in the hyperoxia group compared to the normoxia group (*P* < 0.001, Supplementary Table S2). Although it appears that hyperoxia has a stronger effect on the scotopic ERG responses of the *Dio2 KO* animals, this could be due to an overall lower responses recorded from the *Dio2 KO* animals compared to the control (Figs. 4B, 4B’, 4D). This is evident when the data was replotted by normalizing the responses of the hyperoxia to normoxia between the controls and the *Dio2 KO* groups. As seen in Figure 4B’, the recovery of the bipolar cell response in the *Dio2 KO* hyperoxia animals is accelerated compared to the hyperoxia control group, although this response is not significant. The data suggest that, independent of Dio2 loss, hyperoxia results in long-term damage to the bipolar cells. Additionally, hyperoxia causes some impairment in cone photoreceptor function because photopic responses are reduced in the *Dio2 KO* (*P* < 0.001) compared to the normoxia controls at 0.6, 1.4 and 1.9 log cd ∙ s/m^2^ (Supplementary Table S2, Fig. 4C). Overall, the ERG results indicate that exposure to hyperoxia results in long-term signaling defects either originating from the photoreceptors or due to changes in the bipolar cells, and loss of Dio2 exacerbates these defects.

### Hyperoxia-Induced Cellular Changes are Mildly Persistent in the Adult *Dio2 KO* Animals

**Figure 5.**
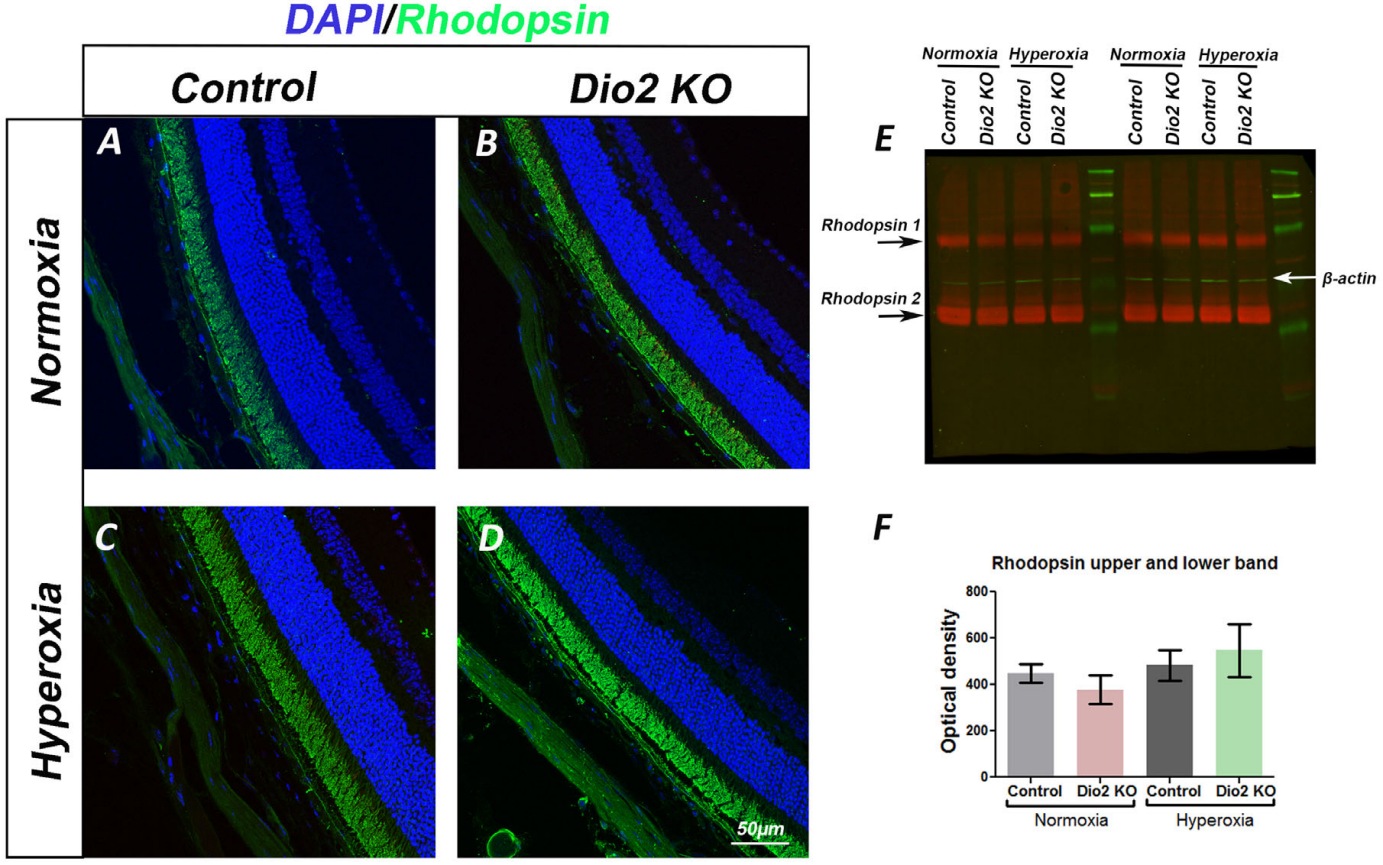
Loss of *Dio2* does not affect rod photoreceptor recovery (**A–D**) Adult retinal sections from control (**A,C**) and *Dio2 KO* (**B,D**) animals immune-labeled with anti-rhodopsin antibody (*green*) and DAPI (*blue*). Retinal sections of animals maintained under normoxia are shown in panels **A, B** and hyperoxia are panels, **C,D**. (**E**) Immunoblot of retinal lysates from adults, probed with rhodopsin antibody (*red*) and β-actin (*green*) as a loading control. *Arrows* indicate the upper and lower bands for the rhodopsin protein that were included in the quantification. (**F**) The relative density of rhodopsin/β-actin protein. Controls are *Dio2^+/^*^−^*.* Four blots were used for the quantitation, and for each blot, retinal lysates from different animals were used. *Error bars:* ±SEM.

**Figure 6.**
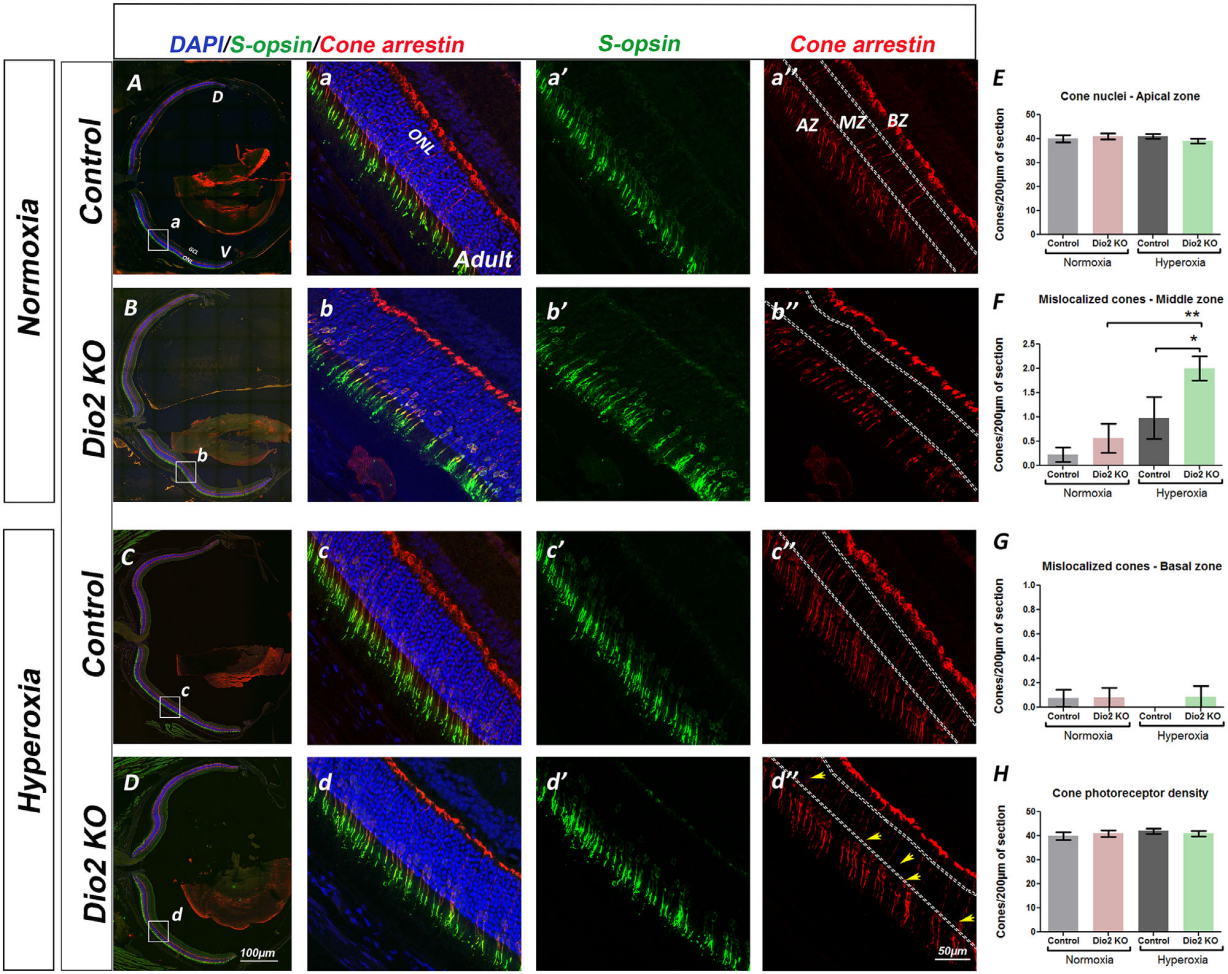
Cone nuclear mislocalization is still persistent in adult *Dio2* KO hyperoxia animals. (**A–D”**) Adult retinal sections stained with S-opsin (*green*), cone arrestin (*red*), DAPI (*blue*) from control (**A–A”** and **C–C”**) and *Dio2 KO* (**B–B”**, **D’–D”**) animals maintained in normoxia (**A–B”**) and animals that were exposed to hyperoxia (**C–D”**). (**E–G**) Graph represents the total number of mislocalized cones quantified in the apical (**E**), middle (**F**), and basal zone (**G**) from the different groups. A significant number of mislocalized cone nuclei can be detected in the middle zone of the *Dio2 KO* adult animals compared to the control (**H**). *Graph* represents the quantitation of the total cone photoreceptor density/ 200 µm of the section. Controls are *Dio2^+/^*^−^. Yellow arrowheads show the mislocalized cones (n = 6). *P < 0.05, **P < 0.01, ***P < 0.001. Data were analyzed using two-way ANOVA, and pairwise post-hoc comparisons were performed using Fisher LSD method. *Error bars:* ± SEM.

The ERG defects in the adults suggest that perhaps the cellular changes observed at P23 are persistent in the adults (seven weeks) and contribute to impaired retinal physiology. We analyzed the adult retina using the same markers that were used at P23. As seen at P23, rhodopsin immunoreactivity appears to be similar between the normoxia and the hyperoxia group within the various genotypes (Figs. 5A–5D). To confirm these results, we performed Western blot analysis on retinal lysates from the various groups and quantitated relative levels of the rhodopsin protein compared to beta-actin. In agreement with the immunofluorescence staining, overall rhodopsin protein levels are similar between the various groups (Figs. 5E, 5F). Thus it is highly unlikely that the origin of the reduced photopic ERG responses is due to a reduction in rhodopsin level or distribution. In agreement with the ERG data, PKC-α immunostaining further confirms our observations that the rod bipolar cells are damaged by hyperoxia (Supplementary Fig. S6). In the hyperoxia control group, the rod bipolar cell dendrites are abnormally extending into the ONL in several areas compared to the normoxia controls (Supplementary Fig. S6C, S6C’). Similarly, but to a lesser extent, dendritic outgrowths could be detected in the *Dio2 KO* hyperoxia retina (Supplementary Figs. S6D, S6D’). These results suggest that hyperoxia severely affects rod bipolar cell morphology and function, but there is no additional effect caused by loss of Dio2. To assess whether the cone photoreceptors are permanently altered by hyperoxia, retinal sections were labeled with S-opsin and Cone arrestin (Figs. 6A–6d”). As seen at P23, there is also a significantly higher number of cone nuclei present in the middle zone of the ONL in the *Dio2 KO* hyperoxia mice at seven weeks (Fig. 6F compared to the *Dio2 KO* normoxia group [*P* = 0.005] and control hyperoxia group [*P* = 0.043] [Figs. 6d”, 6F]). In the control normoxia and hyperoxia groups, the cone nuclei were apically localized, thus suggesting that *Dio2* is required for the correct apical localization of the cone nuclei. We rarely observed any mislocalized cone nuclei in the basal zone for any of the groups. Taken together, these results indicate that hyperoxia-induced cone nuclear mislocalization is still persistent in adult *Dio2 KO* animals. These persistent milder changes in the cone photoreceptor nuclear localization could explain why at higher intensities the scotopic b-wave amplitudes were reduced in the *Dio2 KO* hyperoxia group compared to the control hyperoxia group.

### Independent of *Dio2* Loss, OIR Causes Long-Term Changes in Inner Retinal Physiology

**Figure 7.**
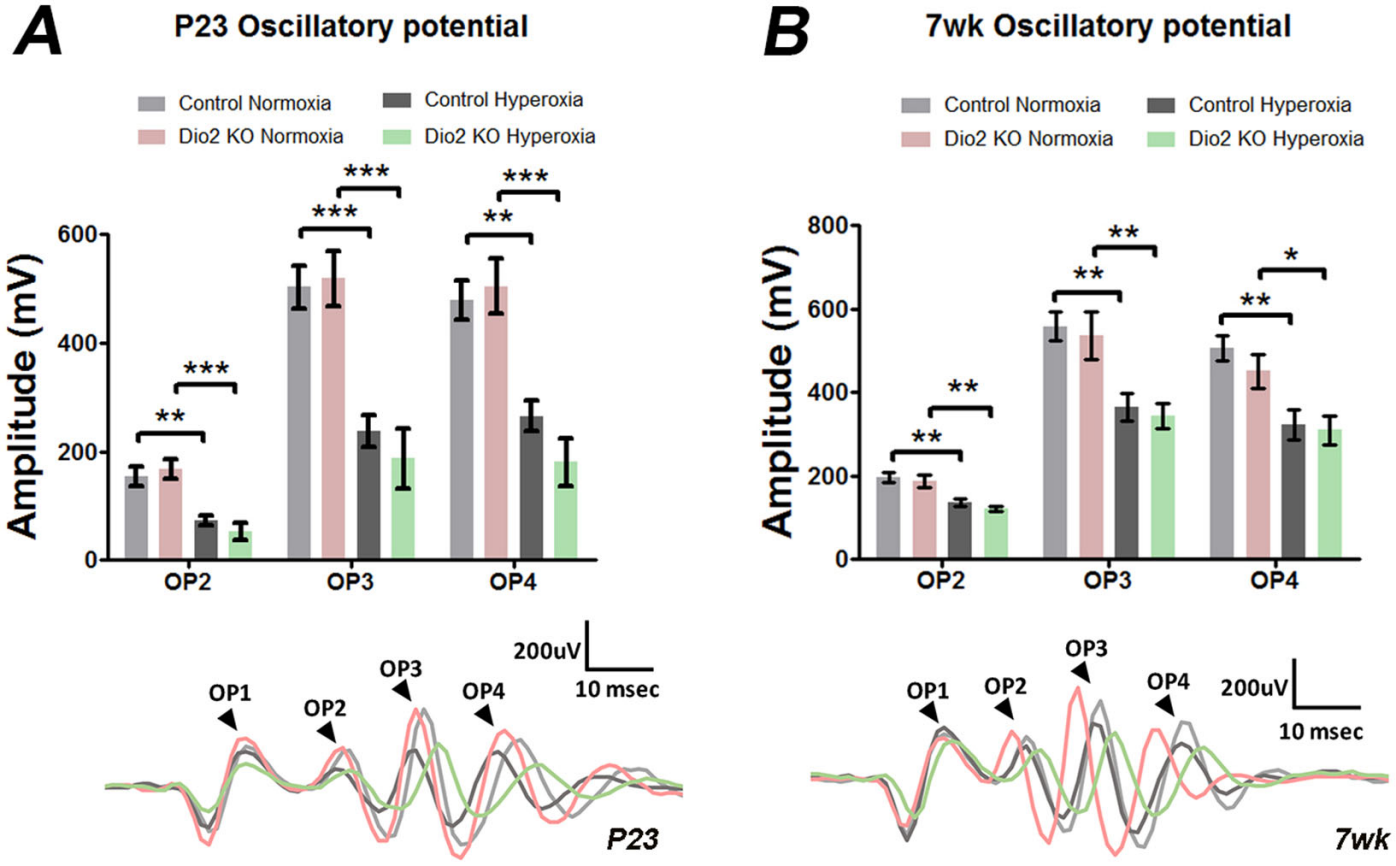
Hyperoxia has long-term effects on inner retinal neuron function. (**A, B**) Amplitudes of filtered oscillatory potentials (OPs), calculated from the recorded ERG responses under scotopic conditions at P23 (**A**) and seven weeks (**B**). Both at P23 and seven weeks the amplitudes of OPs are significantly reduced in the hyperoxia groups compared to the normoxia group irrespective of the genotype of the animals (n = 4–6). *P < 0.05, **P < 0.01, ***P < 0.001. P23 Controls are *Dio2^+/+^ & Dio2^+/^*^−^*,* adult controls are *Dio2^+/^*^−^. Data were analyzed using two-way ANOVA, and pairwise post-hoc comparisons were performed using Tukey's method. *Error bars:* ±SEM.

Hyperoxia is known to cause cellular loss within the inner nuclear layer (INL). To address whether loss of *Dio2* contributes to further changes in the cells of the INL, we measured oscillatory potentials (OPs). OPs are small wavelets on the ascending (depolarizing) part of the b-wave of the ERG. OPs are believed to originate primarily from amacrine cells and ganglion cells with partial contribution from the bipolar cells. OP1 is not reported because it is affected by a-wave responses. OP2 to OP4 were measured at P23 (Fig. 7A) and seven weeks (Fig. 7B) of age. At both time points, OPs were significantly reduced in hyperoxia groups compared to normoxia groups irrespective of the genotype of the animals within that group. Overall, the OP measurements indicate that hyperoxia results in the reduction of OPs, and loss of *Dio2* does not have any significant role in OP wave forms.

## Discussion

We and others have shown that thyroid hormones are very important for photoreceptor development and function.^16^^,^^17^^,^^29^^,^^30^ We have previously reported that thyroid hormone activating enzyme *Dio2* is predominately expressed in the cone photoreceptors.^18^ The OIR model is a commonly used model to study the pathophysiology of ROP. However, most OIR studies are focused on mitigating the effects of the pathological angiogenesis, despite the continued visual impairment in patients treated for ROP or with regressed ROP. This study is focused on investigating the contribution of Dio2/thyroid hormone signaling in neuronal cells using the OIR model. We show that retinal physiology and morphology is severely compromised in the *Dio2 KO* animals at P23, as well as in adults. At P23, all retinal ERG responses are severely compromised in the animals exposed to hyperoxia compared to the normoxia animals. This is not surprising, because others have reported that the neuronal responses from rod and cone pathways are severely altered by hyperoxia.^31^^,^^32^ Our data suggest that the loss of Dio2 further exacerbates these defects, thereby demonstrating that thyroid hormone signaling is an important contributor to the pathophysiology of ROP. Alternatively, the severity of the rod and cone pathway defects in the *Dio2 KO* animals could be due to excessive loss of the vasculature under hyperoxia in the Dio2 mutants. Loss of vasculature will lead to poor perfusion and increased cell death, as well as compromised function. However, loss of vasculature in the hyperoxia animals is similar at P12 between the control and the *Dio2 KO*, thus ruling out the possibility that this phenotype could be due to excessive vascular death in the *Dio2 KO* hyperoxia group (Supplementary Fig. S7).

To address whether the effects of Dio2 loss are transient, animals exposed to hyperoxia from P7 to P12 were returned to normoxia and reassessed for retinal physiology in the adults. Retinal function is compromised in the adult *Dio2 KO* animals exposed to hyperoxia but not in the *Dio2 KO* animals maintained in room air. Surprisingly, in the control hyperoxia animals, though a-wave ERG responses are normal at seven weeks of age, the scotopic b-wave responses appear to be significantly reduced compared to the room air group (Supplementary Table S2). Additionally, in the adult *Dio2 KO* hyperoxia animals photoreceptor responses are still reduced, suggesting that loss of Dio2 continues to have an effect on photoreceptor function.

Reduction in the amplitude of scotopic a-wave is indicative of functional changes in the photoreceptors or alternatively could indicate a loss of rod photoreceptors. Our study indicates that loss of photoreceptors or a reduction in the length of the outer segments is not likely to be the cause of the reduction in a-wave amplitude, because we did not detect a concomitant loss of rhodopsin levels in the *Dio2 KO* mice exposed to hyperoxia. Instead, the reduction in a-wave amplitude is likely due to a structural fault in the photoreceptor outer segments.^31^ It is important to note that the scotopic a-wave responses of the *Dio2 KO* hyperoxia animals are significantly affected (Supplementary Table S2) only at higher flash intensities (≥ 0.6 log cd ∙ s/m^2^) but not at lower flash intensities (≤ 0.0 log cd ∙ s/m^2^). Defect in rod signaling at higher intensities further supports the idea there are structural defects in rods that causes functional breakdown after a certain threshold has been reached. How Dio2/TH signaling regulates rod photoreceptor maturation and whether this is a non-cell autonomous function of Dio2 merits further investigation.

Interestingly, our data also suggest that the dark-adapted b-wave responses are severely compromised in the *Dio2 KO* animals that were exposed to hyperoxia. In contrast to the rod photoreceptors, this response can be attributed to a loss of cells in the INL because several groups have reported a thinning of the INL in animals with OIR.^33^ However, in the hyperoxia *Dio2 KO* animals, there wasn't any obvious decrease in INL thickness compared to the controls (Fig. 2). Although we did not measure INL thickness, it is unlikely that cell loss in the INL is the cause for the compromised b-wave responses at P23. A likely reason is that there is a defect in the bipolar cells or a cumulative effect of the failure in signaling from the photoreceptors to the bipolar cells.^34^ PKC-alpha staining revealed several abnormal dendritic outgrowths into the ONL at P23, both in the control and the *Dio2 KO*. These outgrowths were present in the control retina as well, but the numbers were higher in the *Dio2 KO* animals. Unlike at P23, the b-wave responses recorded in the adults show a reversal of the phenotype. Although not significant, loss of Dio2 appears to aid in the recovery of the bipolar cell responses. Morphologic comparisons of the bipolar cells labeled with PKC-alpha show increased numbers of dendritic outgrowths within the ONL of control animals under hyperoxic conditions. It is not surprising that Dio2 can have opposing effects on retinal development. The main function of Dio2 is to locally control the bioavailability of T3 and thereby regulate T3 mediated signaling. Thyroid hormone signaling needs to be exquisitely controlled both during development and in the adults. Moreover, both increases and decreases in thyroid hormone levels can be detrimental to the cells. In the developing retina, for example, expression of thyroid hormone components is correlated with critical developmental events. Distinct waves of expression correspond with distinct phases of cell proliferation maturation and differentiation.^35^ In mice, increased thyroid hormone signaling can result in cell death of the cone photoreceptors.^36^ In the mature retina, sustained exposure to TH signaling can alter the opsin content. However, specific interrogation of other cell types besides the cones have not be investigated. In patients with hyperthyroidism, studies have shown changes in retinal nerve fiber layers, as well as differences in vasculature.^37^^,^^38^ Thus both increased and decreased thyroid signaling can have context-dependent adverse effects. Our data suggest that Dio2/TH signaling may be required during the development of bipolar cells, but sustained Dio2-mediated TH signaling can be detrimental for the bipolar cells.

Under hyperoxic conditions, although cone numbers were unaffected in the *Dio2 KO* retina, the cone nuclei were mislocalized. Cone photoreceptors are perhaps the best example of polarized nuclear positioning with their nuclei always localized to the apical side whereas their axons extend across the ONL to establish synaptic connections with the second-order neurons. Whether the nuclear positioning confers special function to the cone photoreceptors is unknown. However, several studies have identified the cellular mechanisms underlying the establishment and maintenance of the nuclear spatial confinement. Components of the nuclear envelope proteins are implicated in the anchoring, nuclear migration, or both. Linkers of the nucleoskeleton to the cytoskeleton (LINC complexes) consist of evolutionary-conserved macromolecular assemblies that span the nuclear envelope to connect the nucleus with the peripheral cytoskeleton and are specifically require for cone nuclear positioning. It will be interesting to investigate whether components of this complex are regulated by thyroid hormone signaling. Our study is the first to report a cone nuclear mislocalization in animals exposed to hyperoxia and a role for Dio2 in this process.

It has been reported that hyperoxia can cause disruption in the inner retinal architecture at P17 and in adults as well.^28^^,^^39^ Thus the reduced OP amplitudes could be a result of changes in the numbers of the inner retinal cells. Alternatively, dopamine is known to regulate light-adaptive mechanisms within the retina. Thus a reduction in the oscillatory potential in the OIR model implicates possible functional abnormalities of the dopamine secreting amacrine cells.^40^^−^^42^ Irrespective, the effects on OP amplitudes is not dependent on Dio2 signaling.

The incidence of ROP is strongly correlated with the weight and gestational age at birth. At P23, the *Dio2 KO* animals on average weigh less compared to the control animals (9.4 g vs 6.4 g) when exposed to hyperoxic conditions (Supplementary Fig. S8). However, the *Dio2 KO* animals in normoxia (7.3 g) versus hyperoxia (6.4 g) are not very different in weight. By six weeks of age, there is no difference between the *Dio2 KO* and control animals. Both at P23 and in the adults, the severity of the phenotypes observed in the hyperoxia *Dio2 KO* animals compared to the room air *Dio2 KO* animals suggest that differences in weight is unlikely to be a contributing factor for the impaired retinal physiology and morphological changes.

Retinal dysfunction has been reported in infants with ROP. Our data indicates potential functional impairment in cone photoreceptor signaling, suggesting that children with ROP may have color vision deficits. However, the effect of ROP on color vision in children has contrasting findings with some studies reporting color vision deficits^19^^−^^21^^,^^43^ in infants with severe ROP whereas other studies indicate negative findings.^22^^,^^44^^,^^45^ Nonetheless, similar to our data with the *Dio2 KO* animals, ERG studies have demonstrated delayed cone responses and foveal thinning in children with a history of ROP.^20^^,^^21^^,^^23^^,^^43^^,^^46^ Thyroid hormone deficiency in preterm infants results in reduced contrast sensitivity, slow blue-yellow and red-green color vision processing, suggesting that thyroid hormone levels need to be taken into consideration for ROP development.^47^ Low levels of thyroid hormone are commonly found in the first week after birth, a hormone phenomenon referred to as *transient hypothyroxinemia of prematurity* (THOP). Several trials have treated THOP in extremely low gestational age neonates (24–28 weeks’ gestation). One such study found that the incidence of ROP stage 3 was significantly reduced in infants treated with thyroid hormone versus those in the placebo group. This study further highlights the importance of thyroid hormone treatment for alleviating the effects of ROP.

The goal of our study was to investigate the contribution of local thyroid hormone signaling in pathogenesis of ROP and to determine whether disruption of TH signaling can contribute to long-term vision impairment after recovery from ROP. Our findings illustrate the significance of this signaling axis during acute phases of ROP, as well for the recovery of visual function and warrants further investigation.

## Supplementary Material

Supplement 1

Supplement 2

Supplement 3

Supplement 4

Supplement 5

Supplement 6

Supplement 7

Supplement 8

Supplement 9

Supplement 10
