# Supplementary material for: Thyroid Activating Enzyme, Deiodinase II Is Required for Photoreceptor Function in the Mouse Model of Retinopathy of Prematurity
Source: Invest Ophthalmol Vis Sci. 2020 Nov 25;61(13):36. doi: 10.1167/iovs.61.13.36 (PMC7691789; doi:10.1167/iovs.61.13.36)
Supplement: Supplement 1 [file iovs-61-13-36_s001.pdf]

Figure S1

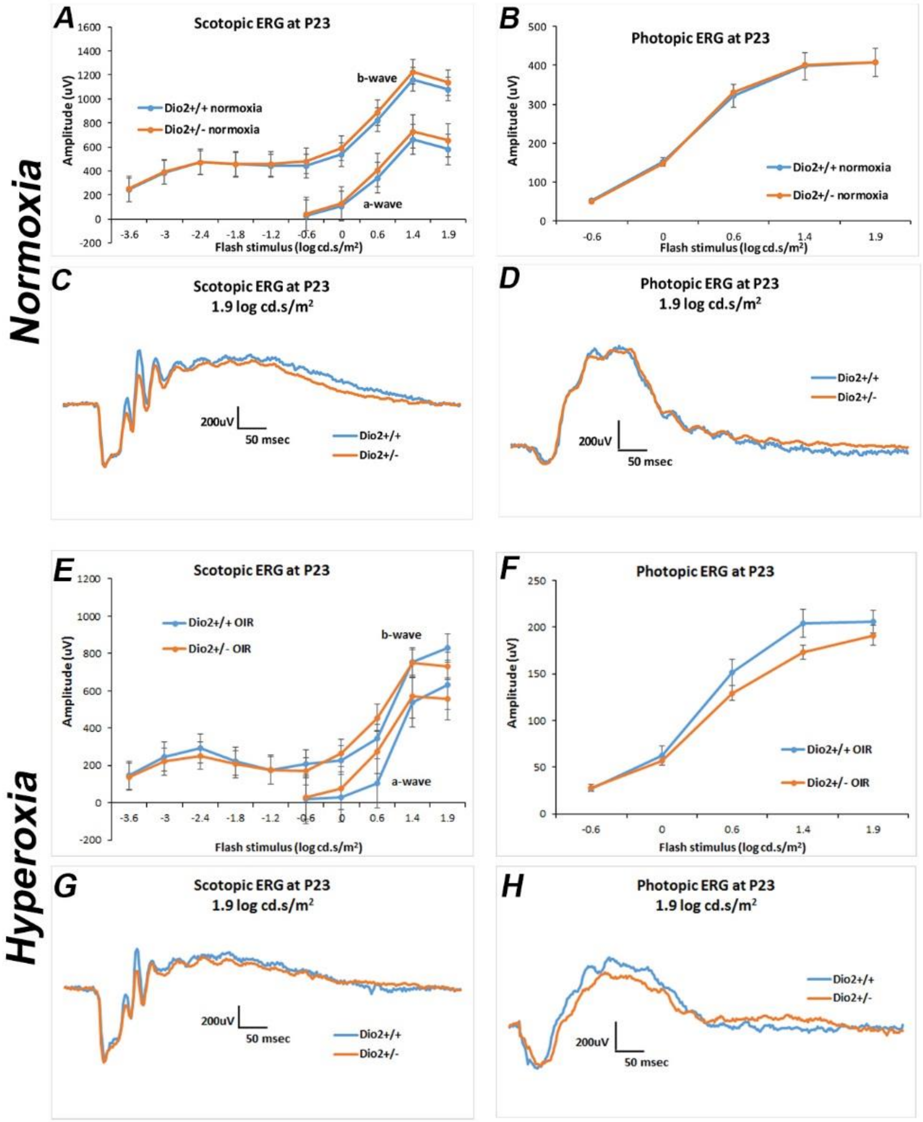

Supplementary Figure 1: Visual responses recorded from the wildtype (*Dio2*$^{+/+}$) and *Dio2* Heterozygote (*Dio2*$^{+/-}$) are indistinguishable: (A, B, E, F) Scotopic and photopic responses recorded from *Dio2*$^{+/+}$ and *Dio2*$^{+/-}$ in normoxia and hyperoxia conditions. (C, D, G, H) Representative ERG waveforms recorded from the *Dio2*$^{+/+}$ and *Dio2*$^{+/-}$ at 1.9 log cd.s/m2 flash illuminance. Control= *Dio2*$^{+/+}$ and *Dio2*$^{+/-}$. n=4.
