# Supplementary material for: Thyroid Activating Enzyme, Deiodinase II Is Required for Photoreceptor Function in the Mouse Model of Retinopathy of Prematurity
Source: Invest Ophthalmol Vis Sci. 2020 Nov 25;61(13):36. doi: 10.1167/iovs.61.13.36 (PMC7691789; doi:10.1167/iovs.61.13.36)
Supplement: Supplement 2 [file iovs-61-13-36_s002.pdf]

Figure S2

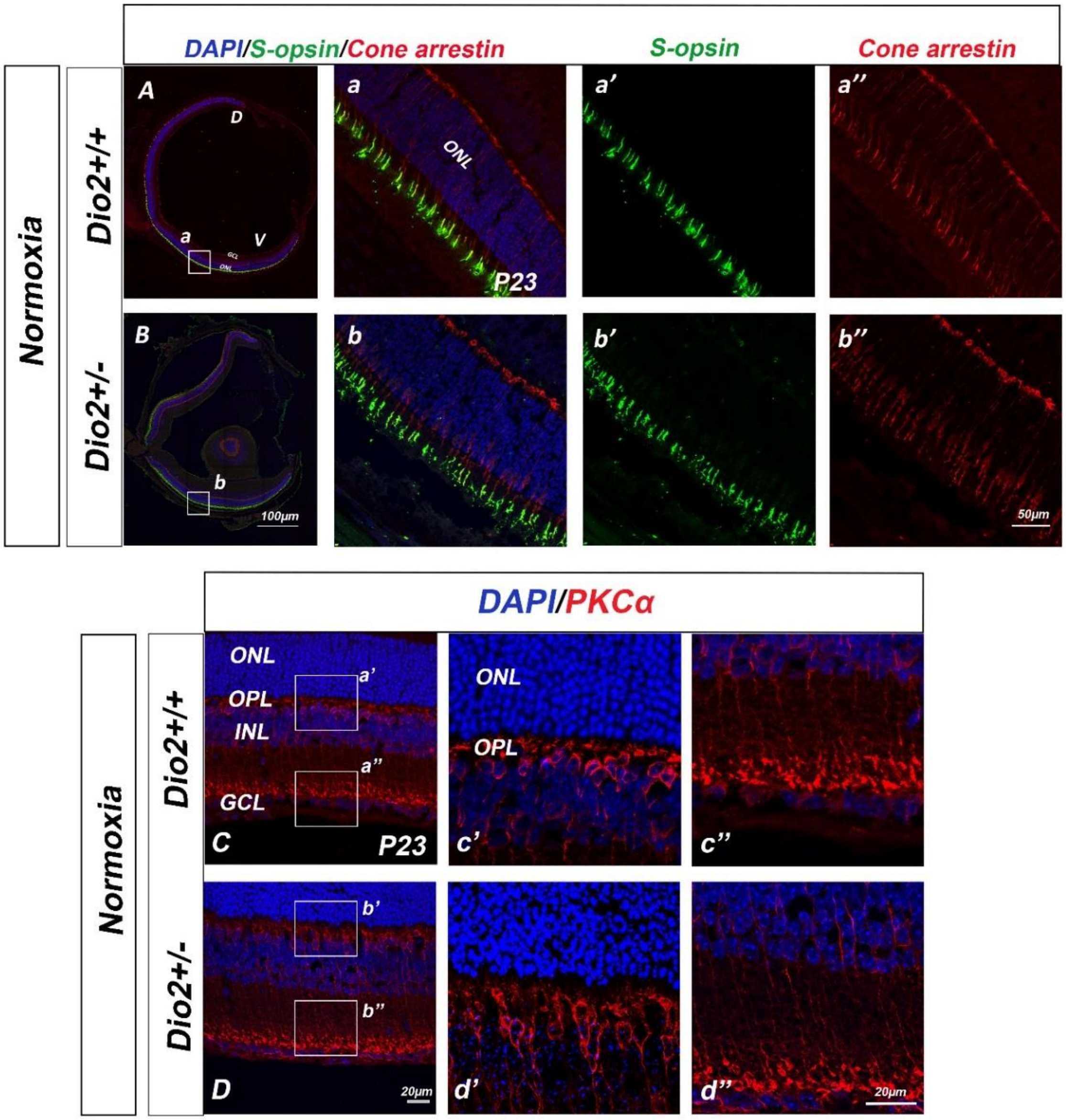

Supplementary Figure 2: Gross morphology and cellular markers are similar between the *Dio2*$^{+/+}$ and *Dio2*$^{+/-}$ animals at P23. (A, B) Cryosections from *Dio2*$^{+/+}$ and *Dio2*$^{+/-}$ animals immunostained with anti S-opsin (green), Cone Arrestin (red) and Dapi (blue). (a-b'') zoomed images of the represented area in white box. (C,D) retinal sections labeled with anti-PKCα (red) and Dapi(blue). (c'-d'') magnified images of the represented areas show in white box. Note that these genotypes are indistinguishable. N=2.
