# Supplementary material for: Thyroid Activating Enzyme, Deiodinase II Is Required for Photoreceptor Function in the Mouse Model of Retinopathy of Prematurity
Source: Invest Ophthalmol Vis Sci. 2020 Nov 25;61(13):36. doi: 10.1167/iovs.61.13.36 (PMC7691789; doi:10.1167/iovs.61.13.36)
Supplement: Supplement 3 [file iovs-61-13-36_s003.pdf]

Figure S3

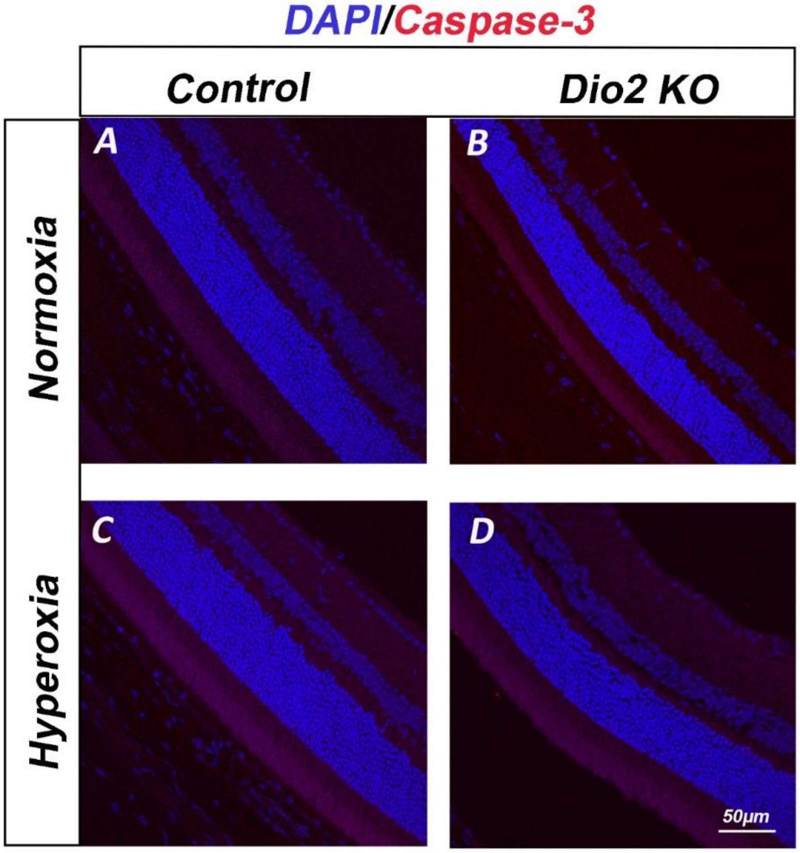

Supplementary Figure 3: Hyperoxia does not result in increased cell death: Immunofluorescence detection of cleaved caspase -3 (red) and DAPI (blue) in the P23 mouse retinal sections. (A, B) Caspase-3 positive cells could not be detected in control and *Dio2 KO* under normoxia and hyperoxia conditions (C, D). Control= $Dio2^{+/+}$ and $Dio2^{+/-}$. n=5.
