# Supplementary material for: Thyroid Activating Enzyme, Deiodinase II Is Required for Photoreceptor Function in the Mouse Model of Retinopathy of Prematurity
Source: Invest Ophthalmol Vis Sci. 2020 Nov 25;61(13):36. doi: 10.1167/iovs.61.13.36 (PMC7691789; doi:10.1167/iovs.61.13.36)
Supplement: Supplement 4 [file iovs-61-13-36_s004.pdf]

Figure S4

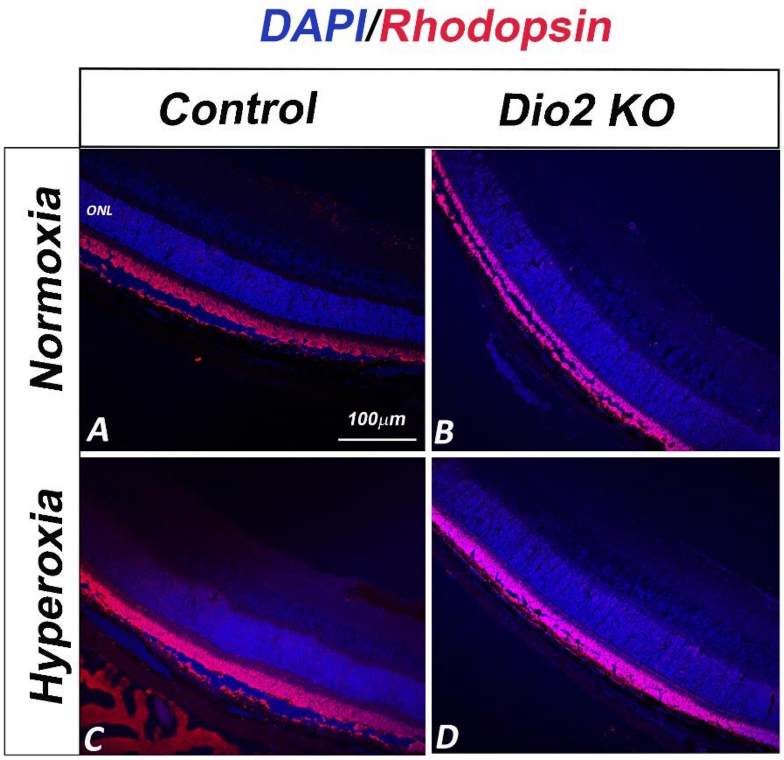

Supplementary Figure 4: Loss of *Dio2* does not affect rod rhodopsin expression (A-D) Retinal sections from P23 control (A,C) and *Dio2 KO* (B,D) animals immunolabeled with anti-rhodopsin antibody (red) and DAPI (blue). Retinal sections from the animals maintained under normoxia are shown in panels A, B and OIR animals are indicated in C, D. Control= *Dio2*$^{+/+}$ and *Dio2*$^{+/-}$. n=5.
