# Supplementary material for: Thyroid Activating Enzyme, Deiodinase II Is Required for Photoreceptor Function in the Mouse Model of Retinopathy of Prematurity
Source: Invest Ophthalmol Vis Sci. 2020 Nov 25;61(13):36. doi: 10.1167/iovs.61.13.36 (PMC7691789; doi:10.1167/iovs.61.13.36)
Supplement: Supplement 5 [file iovs-61-13-36_s005.pdf]

Figure S5

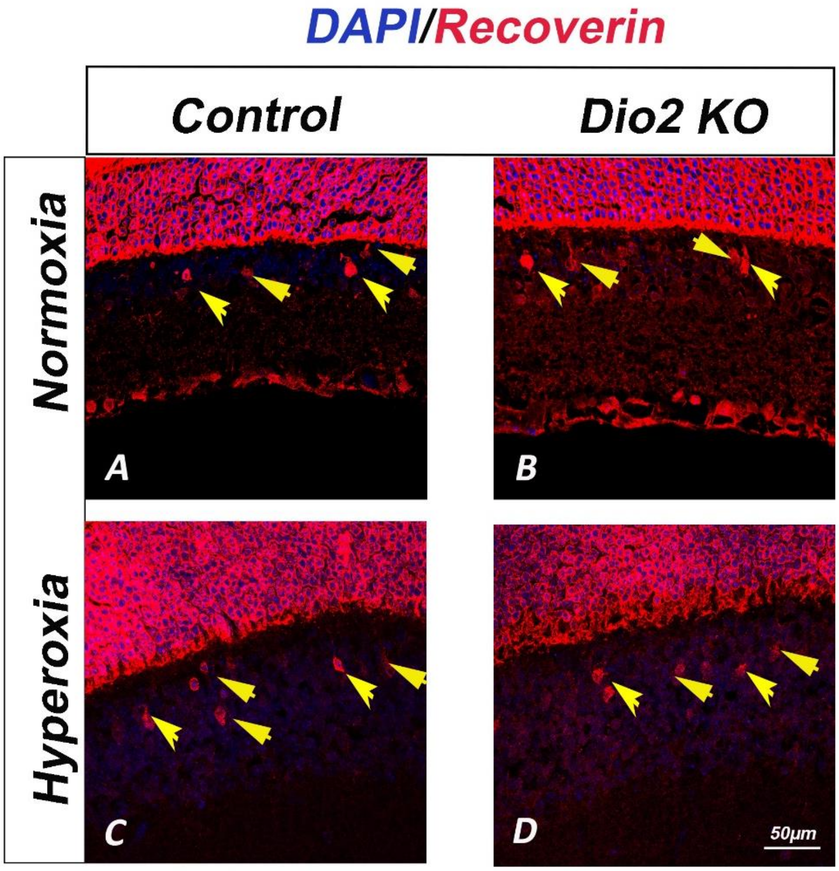

Supplementary Figure 5: Hyperoxia does not alter Recoverin positive cone bipolar cell number: Confocal images of cone bipolar cells stained with Recoverin antibody (red) and DAPI (blue) in the P23 mouse retina. (A, B) Immunolabeling with Recoverin antibody in Control (A, C) and *Dio2 KO* (B,D) under normoxia (A,B) and hyperoxia conditions (C, D). Yellow arrow heads indicate the positive cone bipolar cells. Control = *Dio2*$^{+/+}$ and *Dio2*$^{+/-}$. n=3.
