# Supplementary material for: Thyroid Activating Enzyme, Deiodinase II Is Required for Photoreceptor Function in the Mouse Model of Retinopathy of Prematurity
Source: Invest Ophthalmol Vis Sci. 2020 Nov 25;61(13):36. doi: 10.1167/iovs.61.13.36 (PMC7691789; doi:10.1167/iovs.61.13.36)
Supplement: Supplement 6 [file iovs-61-13-36_s006.pdf]

Figure S6

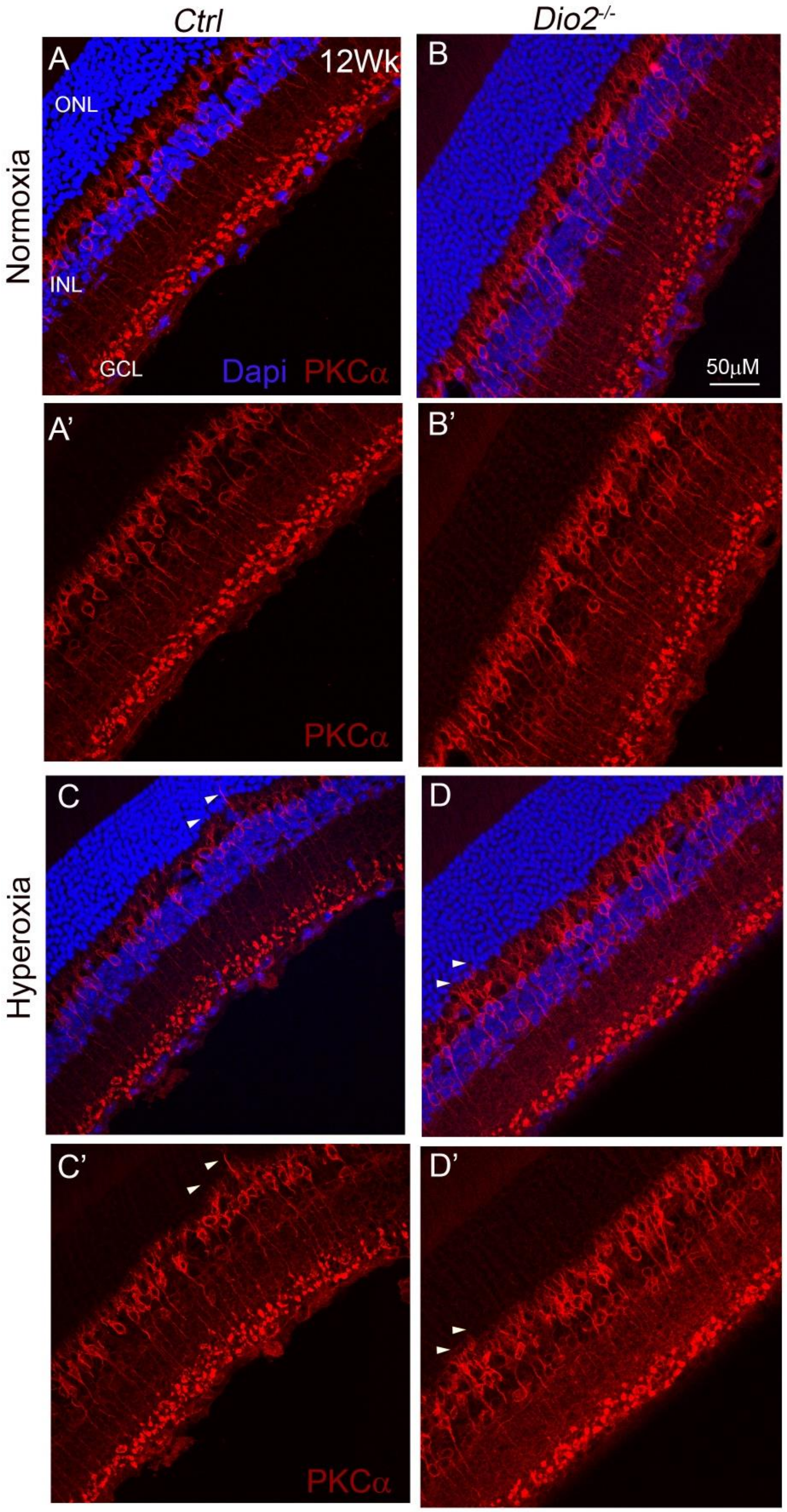

Supplementary Figure 6: Hyperoxia causes permanent changes in Rod bipolar cell morphology in the control and the *Dio2*$^{-/-}$ animals. PKC-α (red) and Dapi (blue) labeling of retinal sections from 12 week old control (*Dio2*$^{+/-}$) and *Dio2*$^{-/-}$ animals that were either kept at room air (A,A',B,B") or in high oxygen from P7-P12 and returned to room air to age till 12 weeks (C,C',D,D'). The rod bipolar cells in the control and the *Dio2*$^{-/-}$ retina appear to extend dendrites in the ONL (white arrowheads), though the phenotype is patchy. Control = *Dio2*$^{+/-}$. n=3.
