# Supplementary material for: Thyroid Activating Enzyme, Deiodinase II Is Required for Photoreceptor Function in the Mouse Model of Retinopathy of Prematurity
Source: Invest Ophthalmol Vis Sci. 2020 Nov 25;61(13):36. doi: 10.1167/iovs.61.13.36 (PMC7691789; doi:10.1167/iovs.61.13.36)
Supplement: Supplement 7 [file iovs-61-13-36_s007.pdf]

Figure S7

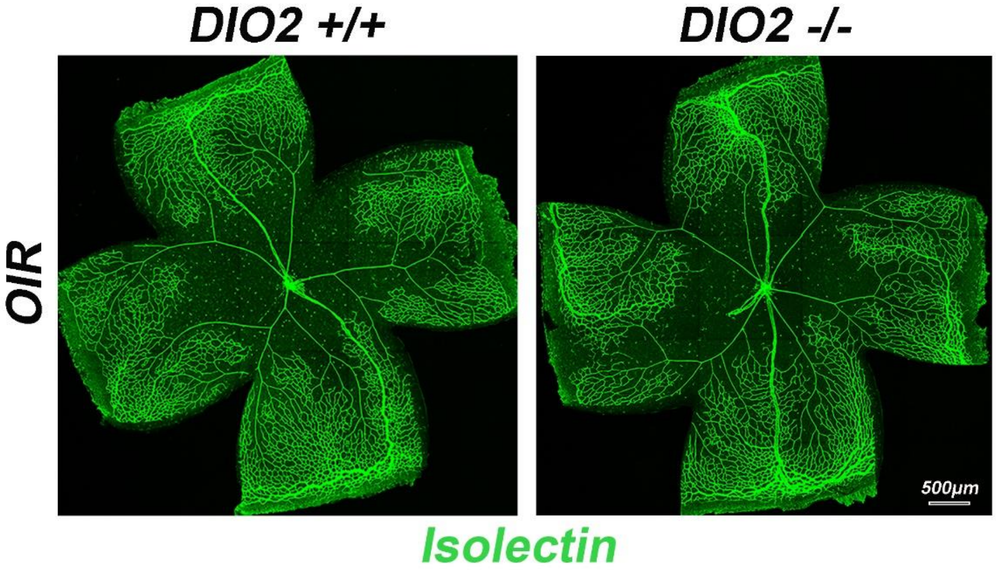

Supplementary Figure 7: Vaso-obliteration in the oxygen-induced retinopathy (OIR) model. Isolectin B4 staining (green) of retinal vasculature in mouse under OIR condition. **Left panel**: the retinal vasculature of a *Dio*$^{+/+}$ mouse at P12 that has been exposed to hyperoxia during P7 to P12. **Right panel:** Retinal vasculature in the *Dio2*$^{-/-}$ mutant mouse. Note the vaso-obliteration in central region of the retina is similar between the control and *Dio2*$^{-/-}$ retina. n=2.
