# Supplementary material for: Thyroid Activating Enzyme, Deiodinase II Is Required for Photoreceptor Function in the Mouse Model of Retinopathy of Prematurity
Source: Invest Ophthalmol Vis Sci. 2020 Nov 25;61(13):36. doi: 10.1167/iovs.61.13.36 (PMC7691789; doi:10.1167/iovs.61.13.36)
Supplement: Supplement 8 [file iovs-61-13-36_s008.pdf]

Figure S8

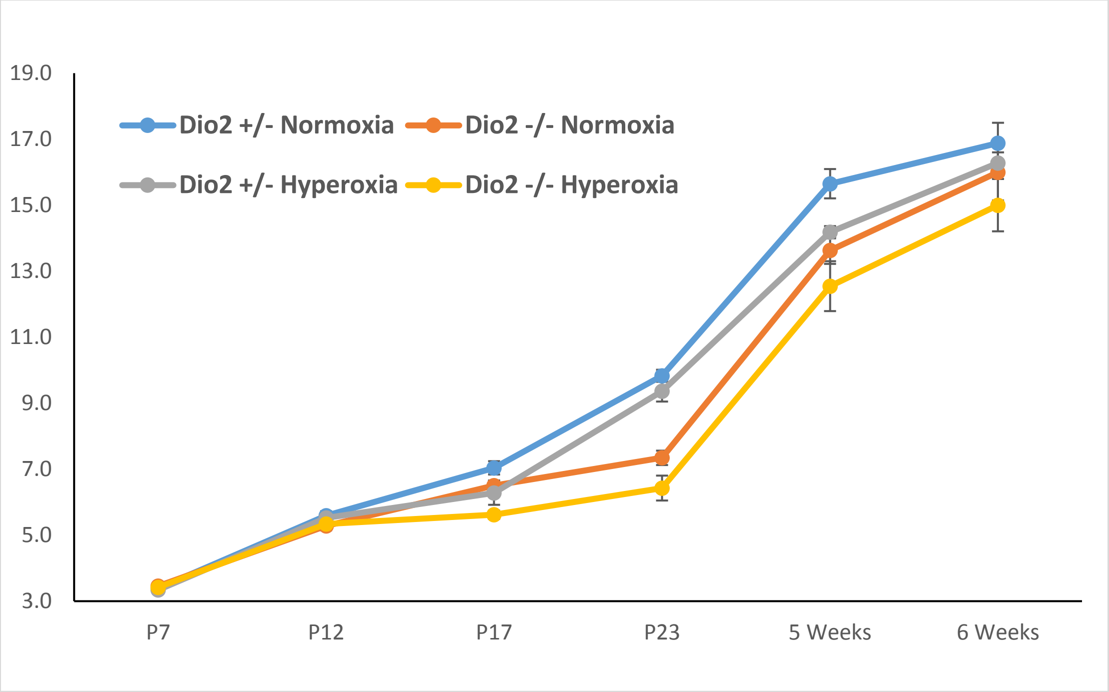

Supplementary Figure 8: Weight gain in animals maintained under normoxia and in hyperoxia. All hyperoxia animals were transferred to the oxygen chamber at P7 and returned to Room Air at P12. Animals were maintained at room air till 6 weeks of age. Prior to hyperoxia exposure, all animals weighed the same but after returning to room air, the weight gain in *Dio2 KO* animals is much lower compared to the controls (9.4gms versus 6.4gm). However, the weight of the *Dio2 KO* hyperoxia animals (6.4gms) is very similar to the *Dio2 KO* animals from room air (7.3gms). Thus exposure to hyperoxia does not affect the weight gain in the *Dio2 KO* animals. n=5-11, error bars are SEM.
