# Supplementary material for: Thyroid Activating Enzyme, Deiodinase II Is Required for Photoreceptor Function in the Mouse Model of Retinopathy of Prematurity
Source: Invest Ophthalmol Vis Sci. 2020 Nov 25;61(13):36. doi: 10.1167/iovs.61.13.36 (PMC7691789; doi:10.1167/iovs.61.13.36)
Supplement: Supplement 9 [file iovs-61-13-36_s009.pdf]

P23 Scotopic a-wave (Figure.1A)

Tukey's multiple comparisons test	Mean Diff.	95.00% CI of diff.	Significant?	Summary	Adjusted P Value
**-2.4**					
Control Normoxia vs. Dio2 KO Normoxia	1.54	-5.488 to 8.569	No	ns	0.884
Control Normoxia vs. Control Hyperoxia	5.969	-2.116 to 14.05	No	ns	0.1728
Control Normoxia vs. Dio2 KO Hyperoxia	6.575	-0.8829 to 14.03	No	ns	0.0885
Dio2 KO Normoxia vs. Control Hyperoxia	4.429	-3.363 to 12.22	No	ns	0.3437
Dio2 KO Normoxia vs. Dio2 KO Hyperoxia	5.035	-2.065 to 12.13	No	ns	0.1909
Control Hyperoxia vs. Dio2 KO Hyperoxia	0.6058	-7.622 to 8.833	No	ns	0.9961
**-1.8**					
Control Normoxia vs. Dio2 KO Normoxia	3.411	-22.46 to 29.28	No	ns	0.9698
Control Normoxia vs. Control Hyperoxia	-2.037	-29.49 to 25.41	No	ns	0.9955
Control Normoxia vs. Dio2 KO Hyperoxia	3.603	-20.51 to 27.72	No	ns	0.9453
Dio2 KO Normoxia vs. Control Hyperoxia	-5.448	-30.33 to 19.43	No	ns	0.9007
Dio2 KO Normoxia vs. Dio2 KO Hyperoxia	0.1911	-19.94 to 20.32	No	ns	>0.9999
Control Hyperoxia vs. Dio2 KO Hyperoxia	5.639	-16.58 to 27.86	No	ns	0.8436
**-1.2**					
Control Normoxia vs. Dio2 KO Normoxia	4.739	-12.32 to 21.79	No	ns	0.7856
Control Normoxia vs. Control Hyperoxia	1.362	-11.67 to 14.39	No	ns	0.9856
Control Normoxia vs. Dio2 KO Hyperoxia	8.14	-9.270 to 25.55	No	ns	0.5083
Dio2 KO Normoxia vs. Control Hyperoxia	-3.377	-19.50 to 12.75	No	ns	0.8694
Dio2 KO Normoxia vs. Dio2 KO Hyperoxia	3.401	-15.36 to 22.16	No	ns	0.939
Control Hyperoxia vs. Dio2 KO Hyperoxia	6.778	-9.388 to 22.94	No	ns	0.5774
**-0.6**					
Control Normoxia vs. Dio2 KO Normoxia	8.119	-29.51 to 45.75	No	ns	0.8467
Control Normoxia vs. Control Hyperoxia	4.785	-16.52 to 26.09	No	ns	0.8983
Control Normoxia vs. Dio2 KO Hyperoxia	23.61	4.205 to 43.02	Yes	*	0.0188
Dio2 KO Normoxia vs. Control Hyperoxia	-3.334	-40.56 to 33.89	No	ns	0.9861
Dio2 KO Normoxia vs. Dio2 KO Hyperoxia	15.49	-22.68 to 53.67	No	ns	0.4566
Control Hyperoxia vs. Dio2 KO Hyperoxia	18.83	0.05150 to 37.60	Yes	*	0.0493
**0**					
Control Normoxia vs. Dio2 KO Normoxia	27.09	-89.29 to 143.5	No	ns	0.8449
Control Normoxia vs. Control Hyperoxia	24.54	-50.50 to 99.57	No	ns	0.6897
Control Normoxia vs. Dio2 KO Hyperoxia	70.6	-17.60 to 158.8	No	ns	0.1296
Dio2 KO Normoxia vs. Control Hyperoxia	-2.549	-121.7 to 116.6	No	ns	0.9997
Dio2 KO Normoxia vs. Dio2 KO Hyperoxia	43.51	-71.77 to 158.8	No	ns	0.6093
Control Hyperoxia vs. Dio2 KO Hyperoxia	46.06	-28.11 to 120.2	No	ns	0.2742
**0.6**					
Control Normoxia vs. Dio2 KO Normoxia	73.46	-200.2 to 347.1	No	ns	0.7637
Control Normoxia vs. Control Hyperoxia	84.53	-65.97 to 235.0	No	ns	0.3301
Control Normoxia vs. Dio2 KO Hyperoxia	223.9	14.55 to 433.3	Yes	*	0.0358
Dio2 KO Normoxia vs. Control Hyperoxia	11.07	-269.6 to 291.7	No	ns	0.9983
Dio2 KO Normoxia vs. Dio2 KO Hyperoxia	150.5	-128.4 to 429.4	No	ns	0.3585
Control Hyperoxia vs. Dio2 KO Hyperoxia	139.4	-56.38 to 335.2	No	ns	0.1845
**1.4**					
Control Normoxia vs. Dio2 KO Normoxia	95.18	-210.4 to 400.7	No	ns	0.7377
Control Normoxia vs. Control Hyperoxia	173.7	-92.02 to 439.3	No	ns	0.1992
Control Normoxia vs. Dio2 KO Hyperoxia	354.4	82.10 to 626.6	Yes	*	0.0134
Dio2 KO Normoxia vs. Control Hyperoxia	78.48	-189.4 to 346.4	No	ns	0.6809
Dio2 KO Normoxia vs. Dio2 KO Hyperoxia	259.2	-7.121 to 525.5	No	ns	0.056

Control Hyperoxia vs. Dio2 KO Hyperoxia	180.7	2.132 to 359.3	Yes	*	0.0472
**1.9**					
Control Normoxia vs. Dio2 KO Normoxia	83.62	-182.9 to 350.2	No	ns	0.7281
Control Normoxia vs. Control Hyperoxia	154.2	-57.19 to 365.7	No	ns	0.1499
Control Normoxia vs. Dio2 KO Hyperoxia	281.9	70.11 to 493.7	Yes	*	0.0135
Dio2 KO Normoxia vs. Control Hyperoxia	70.63	-181.3 to 322.5	No	ns	0.6998
Dio2 KO Normoxia vs. Dio2 KO Hyperoxia	198.3	-48.80 to 445.4	No	ns	0.1043
Control Hyperoxia vs. Dio2 KO Hyperoxia	127.6	7.398 to 247.9	Yes	*	0.0365

P23 Scotopic b-wave (Figure.1B)

Tukey's multiple comparisons test	Mean Diff.	95.00% CI of diff.	Significant?	Summary	Adjusted P Value
**-2.4**					
Control Normoxia vs. Dio2 KO Normoxia	106435	-114371 to 327240	No	ns	0.4381
Control Normoxia vs. Control Hyperoxia	245952	51635 to 440269	Yes	*	0.0197
Control Normoxia vs. Dio2 KO Hyperoxia	357275	162091 to 552460	Yes	**	0.0021
Dio2 KO Normoxia vs. Control Hyperoxia	139517	-53428 to 332463	No	ns	0.1333
Dio2 KO Normoxia vs. Dio2 KO Hyperoxia	250841	62614 to 439067	Yes	*	0.014
Control Hyperoxia vs. Dio2 KO Hyperoxia	111323	-4456 to 227103	No	ns	0.0602
**-1.8**					
Control Normoxia vs. Dio2 KO Normoxia	109088	-117112 to 335287	No	ns	0.4376
Control Normoxia vs. Control Hyperoxia	272056	74506 to 469606	Yes	*	0.0139
Control Normoxia vs. Dio2 KO Hyperoxia	343738	147635 to 539841	Yes	**	0.004
Dio2 KO Normoxia vs. Control Hyperoxia	162968	-37923 to 363859	No	ns	0.0951
Dio2 KO Normoxia vs. Dio2 KO Hyperoxia	234650	38258 to 431042	Yes	*	0.0262
Control Hyperoxia vs. Dio2 KO Hyperoxia	71682	-17681 to 161045	No	ns	0.1328
**-1.2**					
Control Normoxia vs. Dio2 KO Normoxia	123707	-89908 to 337321	No	ns	0.2995
Control Normoxia vs. Control Hyperoxia	304343	101907 to 506779	Yes	**	0.01
Control Normoxia vs. Dio2 KO Hyperoxia	369978	167654 to 572302	Yes	**	0.0042
Dio2 KO Normoxia vs. Control Hyperoxia	180636	16144 to 345128	Yes	*	0.0363
Dio2 KO Normoxia vs. Dio2 KO Hyperoxia	246271	81991 to 410552	Yes	*	0.0116
Control Hyperoxia vs. Dio2 KO Hyperoxia	65635	-7520 to 138791	No	ns	0.0841
**-0.6**					
Control Normoxia vs. Dio2 KO Normoxia	123270	-94991 to 341530	No	ns	0.3172
Control Normoxia vs. Control Hyperoxia	326545	146414 to 506676	Yes	**	0.0042
Control Normoxia vs. Dio2 KO Hyperoxia	380396	201122 to 559670	Yes	**	0.0018
Dio2 KO Normoxia vs. Control Hyperoxia	203275	-4135 to 410686	No	ns	0.0532
Dio2 KO Normoxia vs. Dio2 KO Hyperoxia	257126	52353 to 461900	Yes	*	0.0235
Control Hyperoxia vs. Dio2 KO Hyperoxia	53851	-19668 to 127370	No	ns	0.185
**0**					
Control Normoxia vs. Dio2 KO Normoxia	145745	-156712 to 448203	No	ns	0.4176
Control Normoxia vs. Control Hyperoxia	331366	102870 to 559862	Yes	**	0.0064
Control Normoxia vs. Dio2 KO Hyperoxia	445974	232329 to 659620	Yes	**	0.001
Dio2 KO Normoxia vs. Control Hyperoxia	185621	-106400 to 477642	No	ns	0.2248
Dio2 KO Normoxia vs. Dio2 KO Hyperoxia	300229	4005 to 596454	Yes	*	0.0477
Control Hyperoxia vs. Dio2 KO Hyperoxia	114608	-63807 to 293024	No	ns	0.2677
**0.6**					
Control Normoxia vs. Dio2 KO Normoxia	172215	-280257 to 624687	No	ns	0.5835
Control Normoxia vs. Control Hyperoxia	408754	105369 to 712140	Yes	*	0.0139
Control Normoxia vs. Dio2 KO Hyperoxia	590439	267745 to 913133	Yes	**	0.0014
Dio2 KO Normoxia vs. Control Hyperoxia	236539	-226241 to 699320	No	ns	0.2873
Dio2 KO Normoxia vs. Dio2 KO Hyperoxia	418224	-21971 to 858419	No	ns	0.0607
Control Hyperoxia vs. Dio2 KO Hyperoxia	181685	-54859 to 418228	No	ns	0.1486
**1.4**					
Control Normoxia vs. Dio2 KO Normoxia	201252	-264512 to 667016	No	ns	0.5193
Control Normoxia vs. Control Hyperoxia	505652	112591 to 898713	Yes	*	0.018
Control Normoxia vs. Dio2 KO Hyperoxia	742879	350248 to 1135511	Yes	**	0.002

Dio2 KO Normoxia vs. Control Hyperoxia	304400	-119339 to 728139	No	ns	0.1355
Dio2 KO Normoxia vs. Dio2 KO Hyperoxia	541627	129801 to 953454	Yes	*	0.0167
Control Hyperoxia vs. Dio2 KO Hyperoxia	237228	20180 to 454275	Yes	*	0.0314
**1.9**					
Control Normoxia vs. Dio2 KO Normoxia	172935	-238986 to 584856	No	ns	0.5329
Control Normoxia vs. Control Hyperoxia	475976	156373 to 795579	Yes	**	0.0092
Control Normoxia vs. Dio2 KO Hyperoxia	658061	339311 to 976811	Yes	**	0.0016
Dio2 KO Normoxia vs. Control Hyperoxia	303041	-96594 to 702675	No	ns	0.1142
Dio2 KO Normoxia vs. Dio2 KO Hyperoxia	485126	91354 to 878899	Yes	*	0.0243
Control Hyperoxia vs. Dio2 KO Hyperoxia	182085	22566 to 341604	Yes	*	0.0241

P23 Photopic b-wave (Figure. 1C)

**Tukey's multiple comparisons test**	**Mean Diff.**	**95.00% CI of diff.**	**Significant?**	**Summary**	**Adjusted P Value**
**0.6**					
Control Normoxia vs. Dio2 KO Normoxia	70358	-36000 to 176716	No	ns	0.2079
Control Normoxia vs. Control Hyperoxia	206259	101534 to 310984	Yes	**	0.0032
Control Normoxia vs. Dio2 KO Hyperoxia	243143	139888 to 346398	Yes	***	0.0006
Dio2 KO Normoxia vs. Control Hyperoxia	135901	65267 to 206535	Yes	**	0.0038
Dio2 KO Normoxia vs. Dio2 KO Hyperoxia	172785	100422 to 245148	Yes	***	0.0004
Control Hyperoxia vs. Dio2 KO Hyperoxia	36884	-15695 to 89463	No	ns	0.2008
**1.4**					
Control Normoxia vs. Dio2 KO Normoxia	99772	-31589 to 231132	No	ns	0.138
Control Normoxia vs. Control Hyperoxia	236956	108168 to 365744	Yes	**	0.0043
Control Normoxia vs. Dio2 KO Hyperoxia	283638	156233 to 411043	Yes	***	0.0007
Dio2 KO Normoxia vs. Control Hyperoxia	137184	48103 to 226266	Yes	**	0.0097
Dio2 KO Normoxia vs. Dio2 KO Hyperoxia	183866	91743 to 275990	Yes	**	0.0011
Control Hyperoxia vs. Dio2 KO Hyperoxia	46682	-21809 to 115173	No	ns	0.2173
**1.9**					
Control Normoxia vs. Dio2 KO Normoxia	90910	-39551 to 221370	No	ns	0.1801
Control Normoxia vs. Control Hyperoxia	246356	118742 to 373971	Yes	**	0.0033
Control Normoxia vs. Dio2 KO Hyperoxia	292784	164754 to 420813	Yes	***	0.0005
Dio2 KO Normoxia vs. Control Hyperoxia	155447	67126 to 243768	Yes	**	0.0054
Dio2 KO Normoxia vs. Dio2 KO Hyperoxia	201874	105854 to 297895	Yes	***	0.0006
Control Hyperoxia vs. Dio2 KO Hyperoxia	46427	-30875 to 123730	No	ns	0.3001

Normalized: P23 Scotopic a-wave (Figure.1A')

Bonferroni's multiple comparisons test	Predicted (LS) mean diff.	95.00% CI of diff.	Significant?	Summary	Adjusted P Value
**Dio2 Het - Dio2 KO**					
**-0.6**	0.5601	0.2448 to 0.8754	Yes	****	<0.0001
**0**	0.5834	0.2681 to 0.8987	Yes	****	<0.0001
**0.6**	0.4922	0.1769 to 0.8075	Yes	***	0.0006
**1.4**	0.2945	-0.02082 to 0.6098	No	ns	0.0783
**1.9**	0.2061	-0.1092 to 0.5214	No	ns	0.4274

Normalized: P23 Scotopic b-wave (Figure.1B')

Bonferroni's multiple comparisons test	Predicted (LS) mean diff.	95.00% CI of diff.	Significant?	Summary	Adjusted P Value
**Dio2 Het - Dio2 KO**					
**-3.6**	0.2957	0.04303 to 0.5484	Yes	*	0.0112
**-3**	0.2642	0.01149 to 0.5168	Yes	*	0.034
**-2.4**	0.3031	0.05038 to 0.5557	Yes	**	0.0085
**-1.8**	0.1499	-0.1028 to 0.4026	No	ns	0.9119
**-1.2**	0.1264	-0.1263 to 0.3791	No	ns	>0.9999
**-0.6**	0.08581	-0.1669 to 0.3385	No	ns	>0.9999
**0**	0.2215	-0.03119 to 0.4742	No	ns	0.1341
**0.6**	0.2584	0.005760 to 0.5111	Yes	*	0.0413
**1.4**	0.1998	-0.05291 to 0.4524	No	ns	0.2527
**1.9**	0.1506	-0.1021 to 0.4032	No	ns	0.8983

Normalized: P23 Photopic b-wave (Figure.1C')

Bonferroni's multiple comparisons test	Predicted (LS) mean diff.	95.00% CI of diff.	Significant?	Summary	Adjusted P Value
**Dio2 Het - Dio2 KO**					
0	0.09485	-0.03948 to 0.2292	No	ns	0.2862
0.6	0.1389	0.004529 to 0.2732	Yes	*	0.0401
1.4	0.1371	0.002813 to 0.2715	Yes	*	0.0436
1.9	0.1574	0.02305 to 0.2917	Yes	*	0.0158
