# Supplementary material for: Thyroid Activating Enzyme, Deiodinase II Is Required for Photoreceptor Function in the Mouse Model of Retinopathy of Prematurity
Source: Invest Ophthalmol Vis Sci. 2020 Nov 25;61(13):36. doi: 10.1167/iovs.61.13.36 (PMC7691789; doi:10.1167/iovs.61.13.36)
Supplement: Supplement 10 [file iovs-61-13-36_s010.pdf]

7wk Scotopic a-wave amplitude (Figure. 4A)

Tukey's multiple comparisons test	Mean Diff.	95.00% CI of diff.	Significant?	Summary	Adjusted P Value
**0**					
Control Normoxia vs. Dio2 KO Normoxia	-5852	-85391 to 73688	No	ns	0.9945
Control Normoxia vs. Control Hyperoxia	-5069	-53063 to 42924	No	ns	0.9872
Control Normoxia vs. Dio2 KO Hyperoxia	22380	-28450 to 73209	No	ns	0.6047
Dio2 KO Normoxia vs. Control Hyperoxia	782.8	-80348 to 81913	No	ns	>0.9999
Dio2 KO Normoxia vs. Dio2 KO Hyperoxia	28232	-53518 to 109981	No	ns	0.718
Control Hyperoxia vs. Dio2 KO Hyperoxia	27449	-28376 to 83274	No	ns	0.4984
**0.6**					
Control Normoxia vs. Dio2 KO Normoxia	37496	-82790 to 157781	No	ns	0.7532
Control Normoxia vs. Control Hyperoxia	-1502	-81761 to 78757	No	ns	>0.9999
Control Normoxia vs. Dio2 KO Hyperoxia	96436	15916 to 176957	Yes	*	0.0158
Dio2 KO Normoxia vs. Control Hyperoxia	-38998	-162269 to 84273	No	ns	0.7453
Dio2 KO Normoxia vs. Dio2 KO Hyperoxia	58940	-64062 to 181943	No	ns	0.4866
Control Hyperoxia vs. Dio2 KO Hyperoxia	97938	10301 to 185575	Yes	*	0.027
**1.4**					
Control Normoxia vs. Dio2 KO Normoxia	45836	-129023 to 220695	No	ns	0.8421
Control Normoxia vs. Control Hyperoxia	2688	-107989 to 113365	No	ns	0.9999
Control Normoxia vs. Dio2 KO Hyperoxia	147267	50246 to 244288	Yes	**	0.0028
Dio2 KO Normoxia vs. Control Hyperoxia	-43148	-217516 to 131219	No	ns	0.8473
Dio2 KO Normoxia vs. Dio2 KO Hyperoxia	101431	-70661 to 273523	No	ns	0.278
Control Hyperoxia vs. Dio2 KO Hyperoxia	144579	51567 to 237592	Yes	**	0.0046
**1.9**					
Control Normoxia vs. Dio2 KO Normoxia	46438	-88463 to 181338	No	ns	0.7316
Control Normoxia vs. Control Hyperoxia	4790	-95862 to 105441	No	ns	0.999
Control Normoxia vs. Dio2 KO Hyperoxia	144448	52584 to 236313	Yes	**	0.0022
Dio2 KO Normoxia vs. Control Hyperoxia	-41648	-172012 to 88717	No	ns	0.738
Dio2 KO Normoxia vs. Dio2 KO Hyperoxia	98011	-28886 to 224908	No	ns	0.1336
Control Hyperoxia vs. Dio2 KO Hyperoxia	139658	60459 to 218858	Yes	**	0.0018

7wk Scotopic b-wave amplitude (Figure. 4B)

Tukey's multiple comparisons test	Mean Diff.	95.00% CI of diff.	Significant?	Summary	Adjusted P Value
**0**					
Control Normoxia vs. Dio2 KO Normoxia	57049	-143360 to 257458	No	ns	0.8154
Control Normoxia vs. Control Hyperoxia	302859	137244 to 468474	Yes	**	0.0011
Control Normoxia vs. Dio2 KO Hyperoxia	295902	156585 to 435220	Yes	****	<0.0001
Dio2 KO Normoxia vs. Control Hyperoxia	245810	34596 to 457024	Yes	*	0.0235
Dio2 KO Normoxia vs. Dio2 KO Hyperoxia	238853	40684 to 437023	Yes	*	0.0192
Control Hyperoxia vs. Dio2 KO Hyperoxia	-6957	-168946 to 155033	No	ns	0.9991
**0.6**					
Control Normoxia vs. Dio2 KO Normoxia	143423	-62270 to 349117	No	ns	0.2031
Control Normoxia vs. Control Hyperoxia	317460	163869 to 471052	Yes	***	0.0003
Control Normoxia vs. Dio2 KO Hyperoxia	411176	260229 to 562124	Yes	****	<0.0001
Dio2 KO Normoxia vs. Control Hyperoxia	174037	-36105 to 384179	No	ns	0.1095
Dio2 KO Normoxia vs. Dio2 KO Hyperoxia	267753	59705 to 475800	Yes	*	0.0124
Control Hyperoxia vs. Dio2 KO Hyperoxia	93716	-64525 to 251957	No	ns	0.3402
**1.4**					
Control Normoxia vs. Dio2 KO Normoxia	164806	-108585 to 438197	No	ns	0.3197
Control Normoxia vs. Control Hyperoxia	321782	107130 to 536434	Yes	**	0.0036
Control Normoxia vs. Dio2 KO Hyperoxia	465638	276606 to 654671	Yes	****	<0.0001
Dio2 KO Normoxia vs. Control Hyperoxia	156976	-107135 to 421087	No	ns	0.3004
Dio2 KO Normoxia vs. Dio2 KO Hyperoxia	300832	47008 to 554656	Yes	*	0.0237
Control Hyperoxia vs. Dio2 KO Hyperoxia	143856	-25529 to 313241	No	ns	0.0964
**1.9**					
Control Normoxia vs. Dio2 KO Normoxia	147342	-90886 to 385571	No	ns	0.2982
Control Normoxia vs. Control Hyperoxia	318637	126422 to 510852	Yes	**	0.0017
Control Normoxia vs. Dio2 KO Hyperoxia	460605	291342 to 629867	Yes	****	<0.0001
Dio2 KO Normoxia vs. Control Hyperoxia	171295	-64432 to 407022	No	ns	0.1735
Dio2 KO Normoxia vs. Dio2 KO Hyperoxia	313263	89561 to 536964	Yes	**	0.0091
Control Hyperoxia vs. Dio2 KO Hyperoxia	141968	-22732 to 306668	No	ns	0.0949

7wk Photopic b-wave amplitude (Figure. 4C)

Tukey's multiple comparisons test	Mean Diff.	95.00% CI of diff.	Significant?	Summary	Adjusted P Value
**0.6**					
Control Normoxia vs. Dio2 KO Normoxia	46657	-74815 to 168128	No	ns	0.6441
Control Normoxia vs. Control Hyperoxia	87246	-8245 to 182738	No	ns	0.0762
Control Normoxia vs. Dio2 KO Hyperoxia	134766	65197 to 204335	Yes	***	0.0003
Dio2 KO Normoxia vs. Control Hyperoxia	40589	-86144 to 167323	No	ns	0.7481
Dio2 KO Normoxia vs. Dio2 KO Hyperoxia	88109	-30774 to 206992	No	ns	0.1469
Control Hyperoxia vs. Dio2 KO Hyperoxia	47520	-41828 to 136867	No	ns	0.3407
**1.4**					
Control Normoxia vs. Dio2 KO Normoxia	69673	-62329 to 201676	No	ns	0.418
Control Normoxia vs. Control Hyperoxia	95010	-26734 to 216754	No	ns	0.1422
Control Normoxia vs. Dio2 KO Hyperoxia	168242	79154 to 257331	Yes	***	0.0005
Dio2 KO Normoxia vs. Control Hyperoxia	25337	-116120 to 166794	No	ns	0.9416
Dio2 KO Normoxia vs. Dio2 KO Hyperoxia	98569	-25793 to 222931	No	ns	0.1214
Control Hyperoxia vs. Dio2 KO Hyperoxia	73232	-40400 to 186865	No	ns	0.2162
**1.9**					
Control Normoxia vs. Dio2 KO Normoxia	65906	-65098 to 196911	No	ns	0.4602
Control Normoxia vs. Control Hyperoxia	93242	-12463 to 198948	No	ns	0.0904
Control Normoxia vs. Dio2 KO Hyperoxia	168546	77912 to 259180	Yes	***	0.0007
Dio2 KO Normoxia vs. Control Hyperoxia	27336	-101366 to 156038	No	ns	0.9046
Dio2 KO Normoxia vs. Dio2 KO Hyperoxia	102639	-19351 to 224630	No	ns	0.098
Control Hyperoxia vs. Dio2 KO Hyperoxia	75303	-11954 to 162560	No	ns	0.0903

Normalized: 7wk Scotopic a-wave (Figure. 4A')

Bonferroni's multiple comparisons test	Predicted (LS) mean diff.	95.00% CI of diff.	Significant?	Summary	Adjusted P Value
Dio2 Het - Dio2 KO					
-0.6	0.1732	-0.1104 to 0.4568	No	ns	0.5513
0	0.1857	-0.09789 to 0.4693	No	ns	0.4367
0.6	0.1355	-0.1481 to 0.4191	No	ns	>0.9999
1.4	0.1547	-0.1289 to 0.4383	No	ns	0.7658
1.9	0.1634	-0.1202 to 0.4470	No	ns	0.6583
Dio2 Het - Dio2 KO	Predicted (LS) mean 1	Predicted (LS) mean 2	Predicted (LS) mean diff.	SE of diff.	t
-0.6	0.9662	0.793	0.1732	0.1071	1.617
0	1.029	0.8434	0.1857	0.1071	1.734
0.6	1.003	0.8676	0.1355	0.1071	1.265
1.4	0.9961	0.8414	0.1547	0.1071	1.444
1.9	0.9923	0.8289	0.1634	0.1071	1.525

Normalized:7wk Scotopic b-wave (Figure. 4B')

Bonferroni's multiple comparisons test	Predicted (LS) mean diff.	95.00% CI of diff.	Significant?	Summary	Adjusted P Value
Dio2 Het - Dio2 KO					
-3.6	-0.03325	-0.2467 to 0.1802	No	ns	>0.9999
-3	-0.03572	-0.2491 to 0.1777	No	ns	>0.9999
-2.4	-0.02612	-0.2395 to 0.1873	No	ns	>0.9999
-1.8	-0.07462	-0.2880 to 0.1388	No	ns	>0.9999
-1.2	-0.1169	-0.3303 to 0.09654	No	ns	>0.9999
-0.6	-0.1196	-0.3331 to 0.09377	No	ns	>0.9999
0	-0.05966	-0.2731 to 0.1538	No	ns	>0.9999
0.6	-0.008534	-0.2219 to 0.2049	No	ns	>0.9999
1.4	0.01719	-0.1962 to 0.2306	No	ns	>0.9999
1.9	0.03158	-0.1818 to 0.2450	No	ns	>0.9999
Dio2 Het - Dio2 KO	Predicted (LS) mean 1	Predicted (LS) mean 2	Predicted (LS) mean diff.	SE of diff.	t
-3.6	0.6485	0.6817	-0.03325	0.07482	0.4444
-3	0.6647	0.7004	-0.03572	0.07482	0.4774
-2.4	0.601	0.6271	-0.02612	0.07482	0.3491
-1.8	0.5172	0.5919	-0.07462	0.07482	0.9973
-1.2	0.4636	0.5804	-0.1169	0.07482	1.562
-0.6	0.484	0.6036	-0.1196	0.07482	1.599
0	0.589	0.6487	-0.05966	0.07482	0.7973
0.6	0.71	0.7186	-0.008534	0.07482	0.1141
1.4	0.7538	0.7366	0.01719	0.07482	0.2297
1.9	0.7362	0.7046	0.03158	0.07482	0.4221

Normalized: 7wk Photopic b-wave (Figure. 4C')

Bonferroni's multiple comparisons test	Predicted (LS) mean diff.	95.00% CI of diff.	Significant?	Summary	Adjusted P Value
Dio2 Het - Dio2 KO					
-0.6	-0.009385	-0.2258 to 0.2070	No	ns	>0.9999
0	-0.0279	-0.2443 to 0.1885	No	ns	>0.9999
0.6	0.04821	-0.1682 to 0.2646	No	ns	>0.9999

1.4	0.06169	-0.1547 to 0.2781	No	ns	>0.9999
1.9	0.07666	-0.1397 to 0.2930	No	ns	>0.9999
Dio2 Het - Dio2 KO	Predicted (LS) mean 1	Predicted (LS) mean 2	Predicted (LS) mean diff.	SE of diff.	t
-0.6	0.6585	0.6679	-0.009385	0.08172	0.1148
0	0.6635	0.6914	-0.0279	0.08172	0.3414
0.6	0.7313	0.683	0.04821	0.08172	0.5899
1.4	0.7609	0.6992	0.06169	0.08172	0.7549
1.9	0.7625	0.6859	0.07666	0.08172	0.9381
